# Supplementary figures and images for: Characterisation of an Escherichia coli line that completely lacks ribonucleotide reduction yields insights into the evolution of parasitism and endosymbiosis
Source: eLife. 2023 Apr 6;12:e83845. doi: 10.7554/eLife.83845 (PMC10121223; doi:10.7554/eLife.83845)

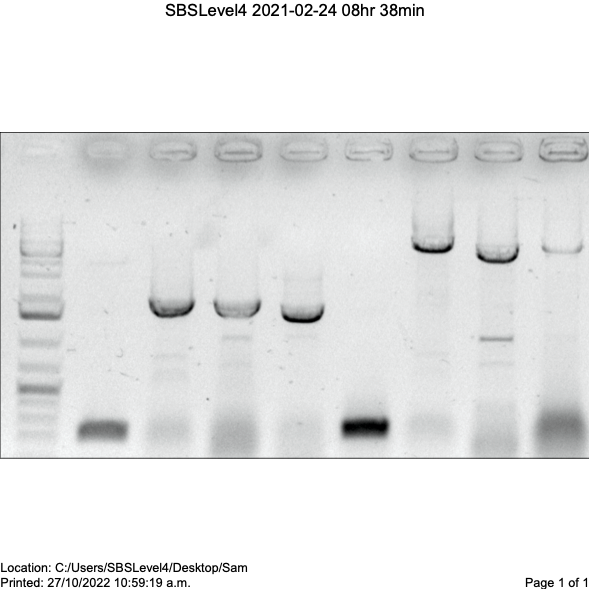

Supplement: Figure 1—source data 1. [file elife-83845-fig1-data1.zip › Figure_1_source_data/Figure 1 source data 1.tif]

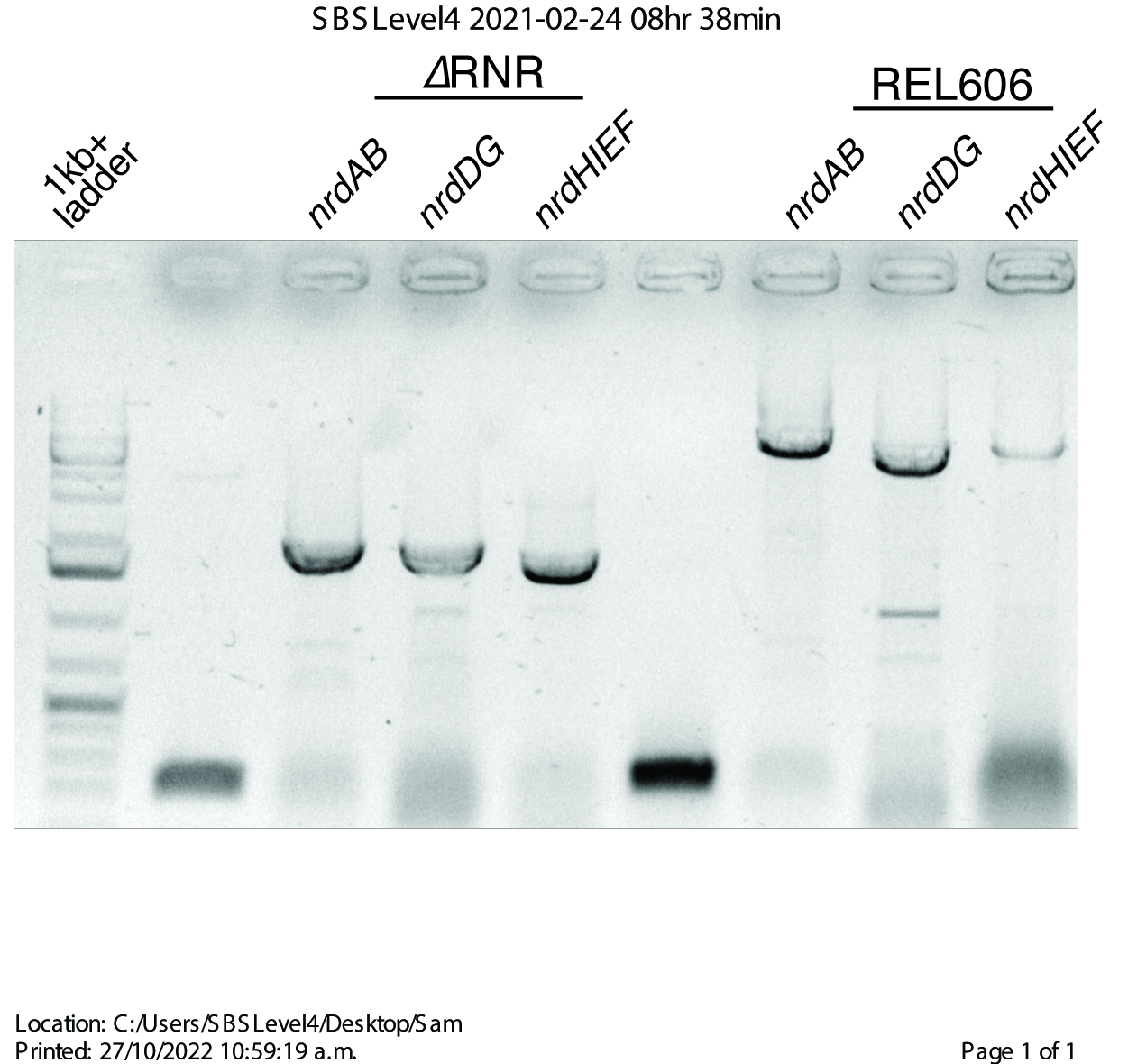

Supplement: Figure 1—source data 1. [file elife-83845-fig1-data1.zip › Figure_1_source_data/Figure 1 source data 2.tif]

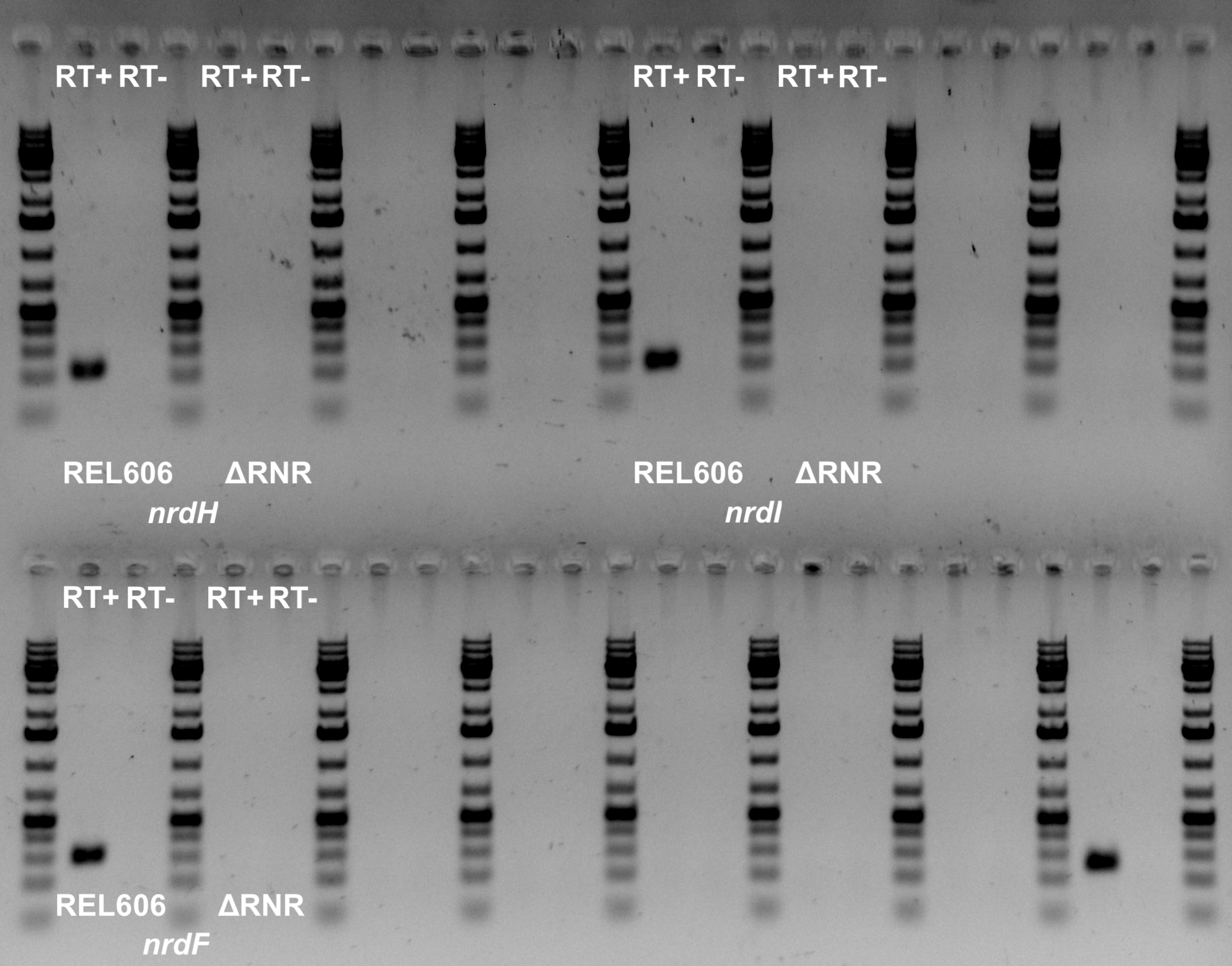

Supplement: Figure 1—figure supplement 1—source data 1. [file elife-83845-fig1-figsupp1-data1.zip › Figure_1-figure_supplement1_source_data/Figure_1-figure_supplement_source_data_4.tif]

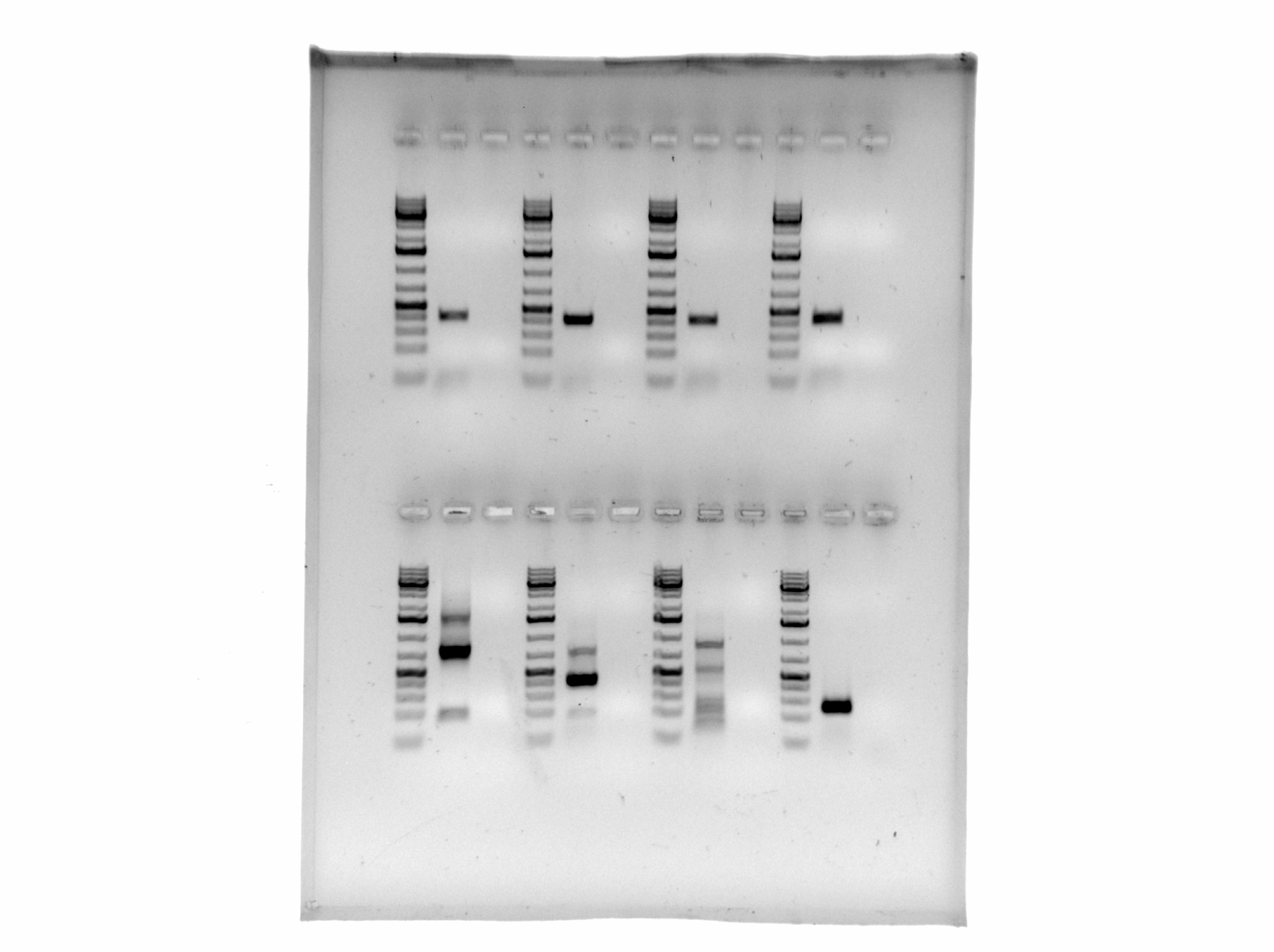

Supplement: Figure 1—figure supplement 1—source data 1. [file elife-83845-fig1-figsupp1-data1.zip › Figure_1-figure_supplement1_source_data/Figure_1-figure_supplement_source_data_5.tif]

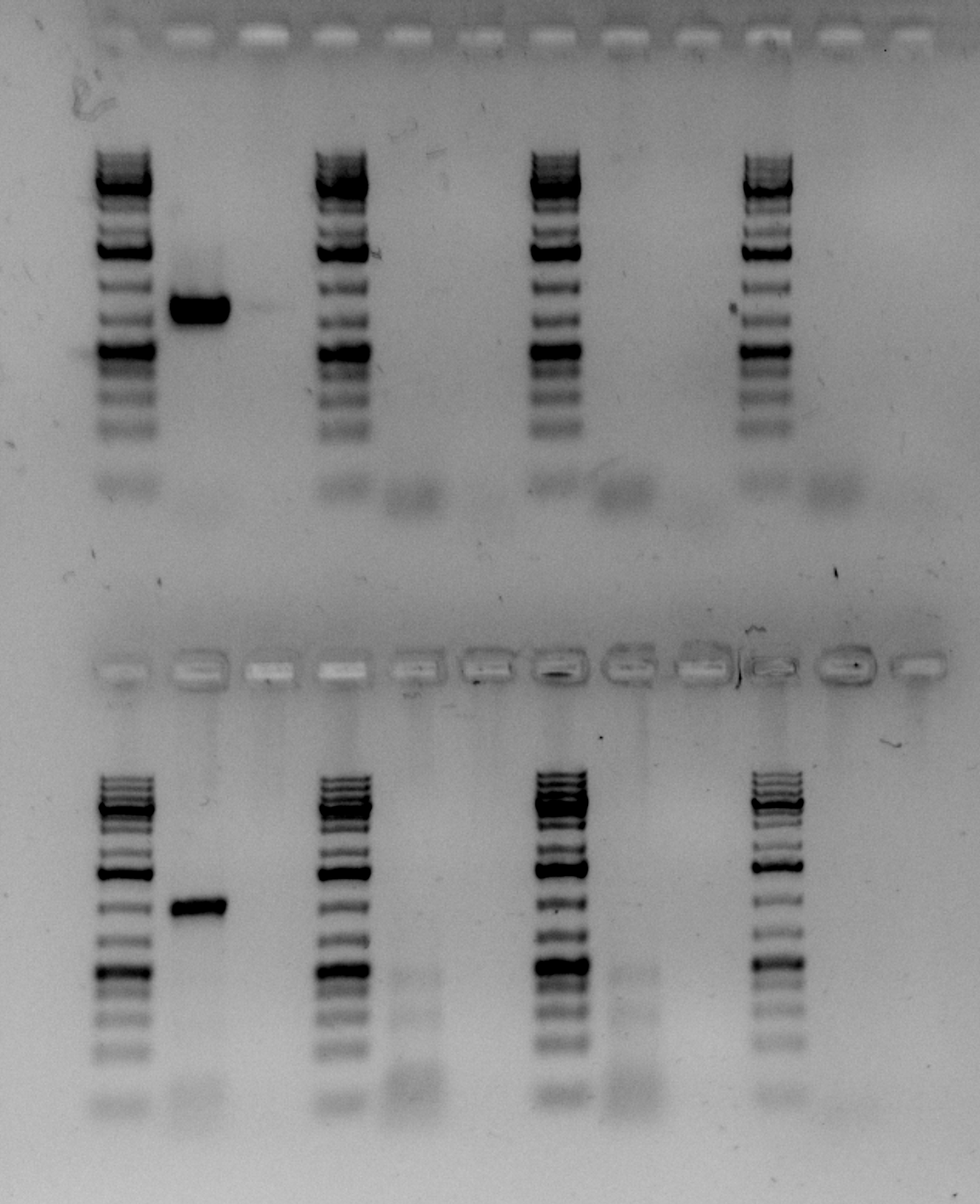

Supplement: Figure 1—figure supplement 1—source data 1. [file elife-83845-fig1-figsupp1-data1.zip › Figure_1-figure_supplement1_source_data/Figure_1-figure_supplement_source_data_7.tif]

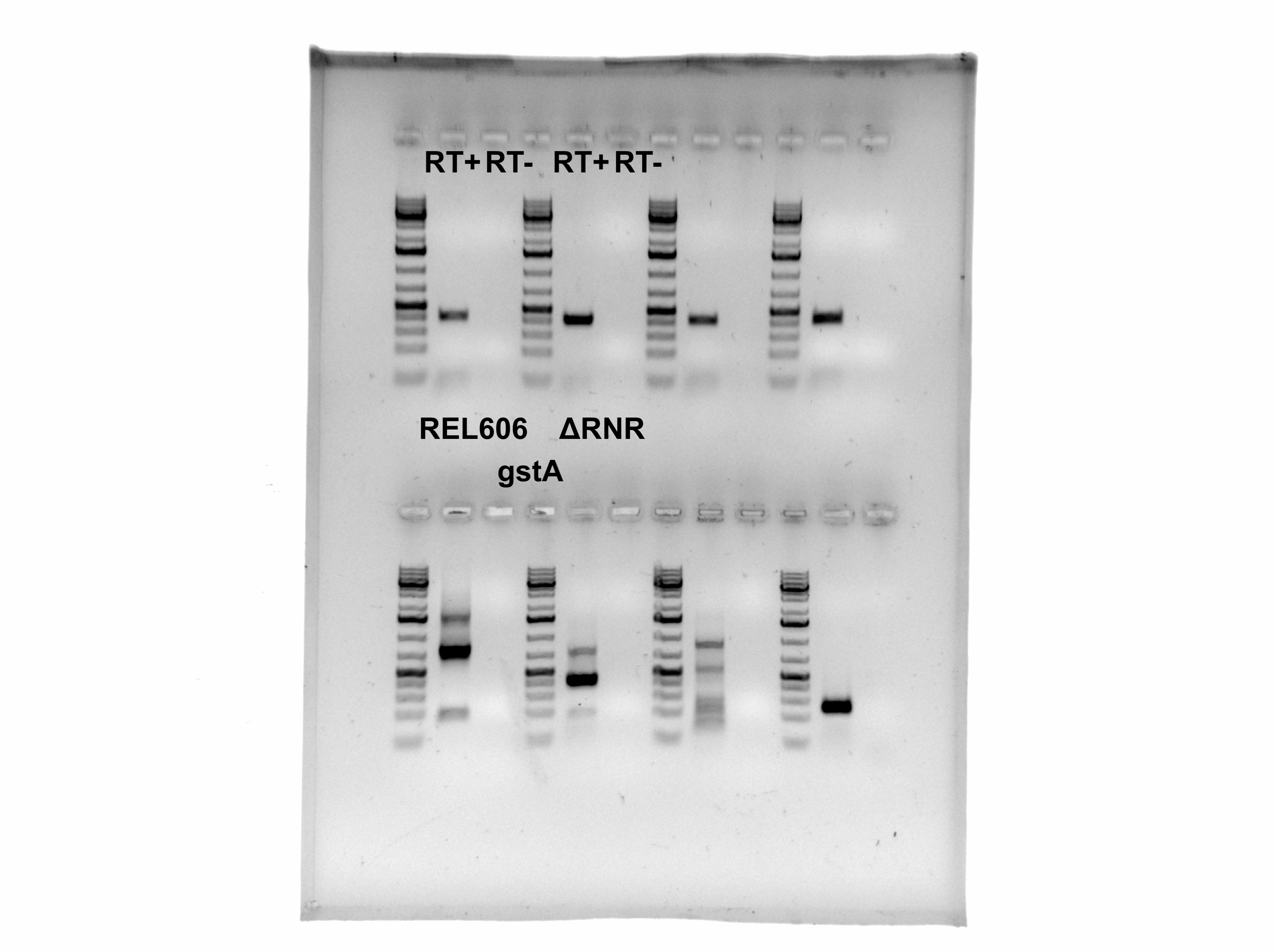

Supplement: Figure 1—figure supplement 1—source data 1. [file elife-83845-fig1-figsupp1-data1.zip › Figure_1-figure_supplement1_source_data/Figure_1-figure_supplement_source_data_6.tif]

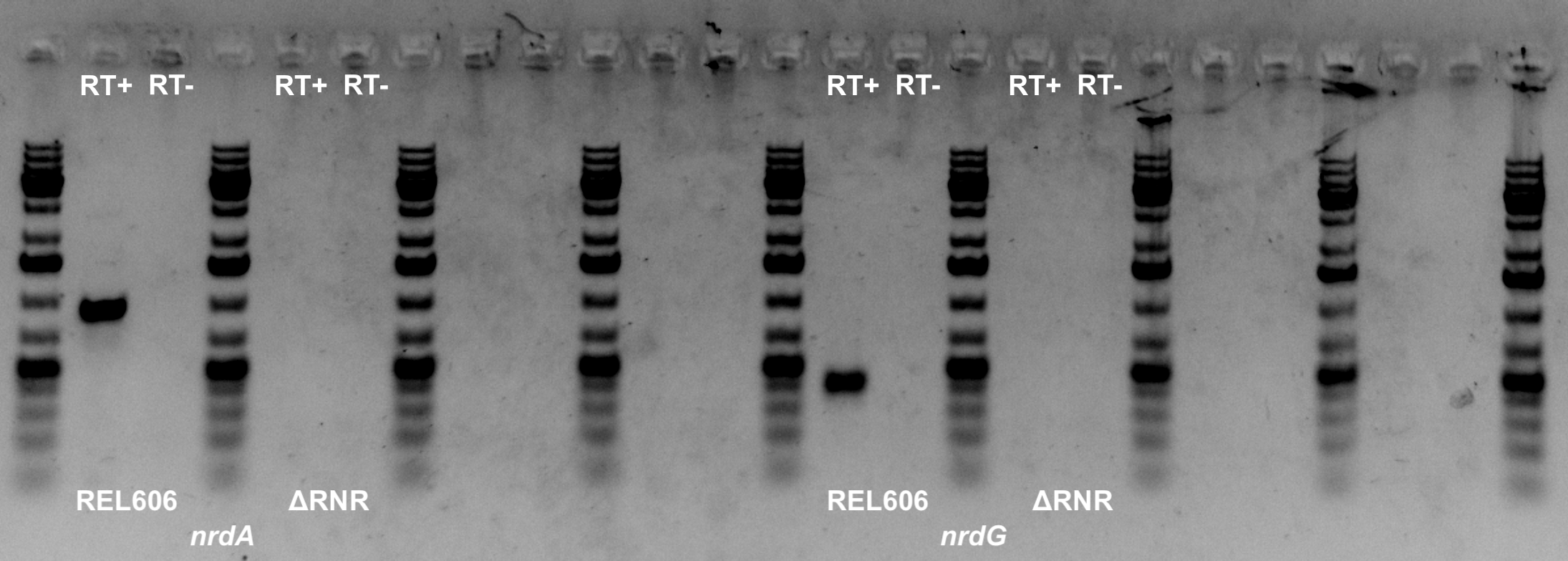

Supplement: Figure 1—figure supplement 1—source data 1. [file elife-83845-fig1-figsupp1-data1.zip › Figure_1-figure_supplement1_source_data/Figure_1-figure_supplement_source_data_2.tif]

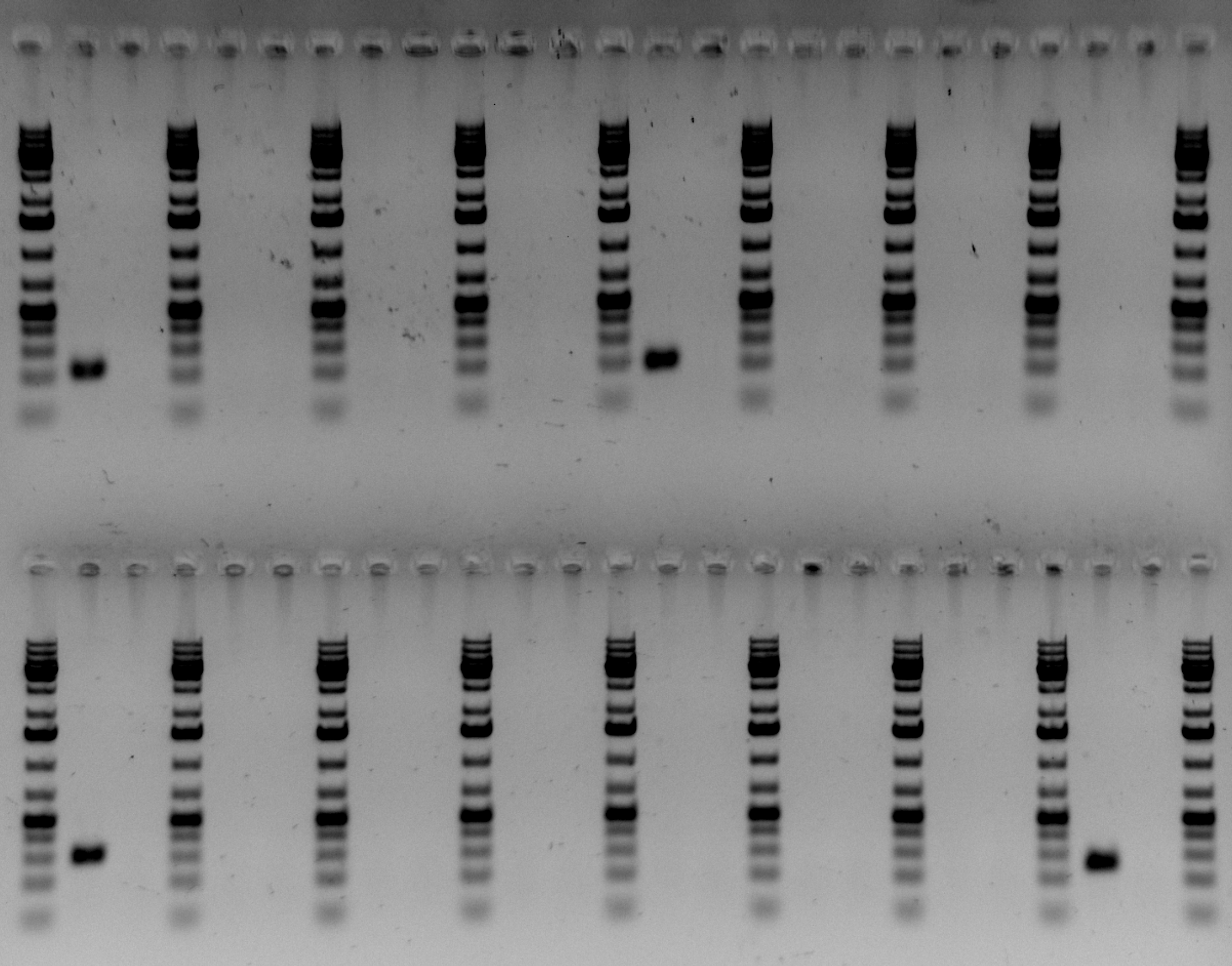

Supplement: Figure 1—figure supplement 1—source data 1. [file elife-83845-fig1-figsupp1-data1.zip › Figure_1-figure_supplement1_source_data/Figure_1-figure_supplement_source_data_3.tif]

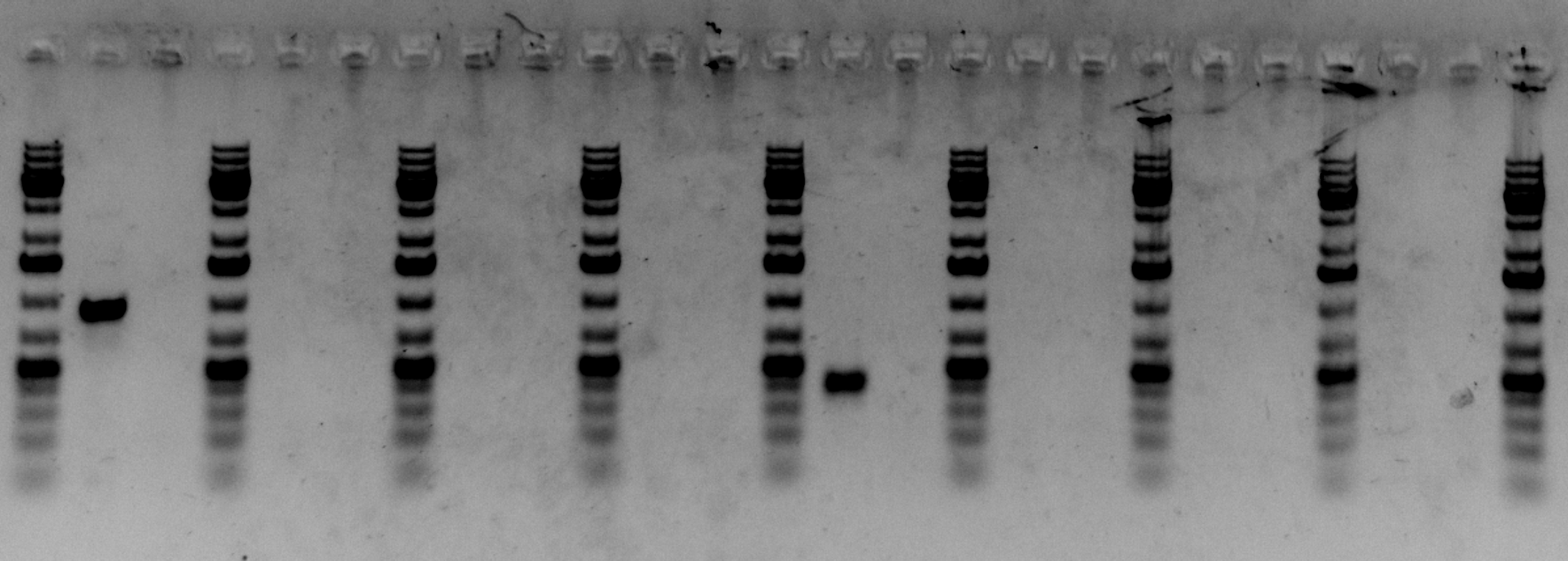

Supplement: Figure 1—figure supplement 1—source data 1. [file elife-83845-fig1-figsupp1-data1.zip › Figure_1-figure_supplement1_source_data/Figure_1-figure_supplement_source_data_1.tif]

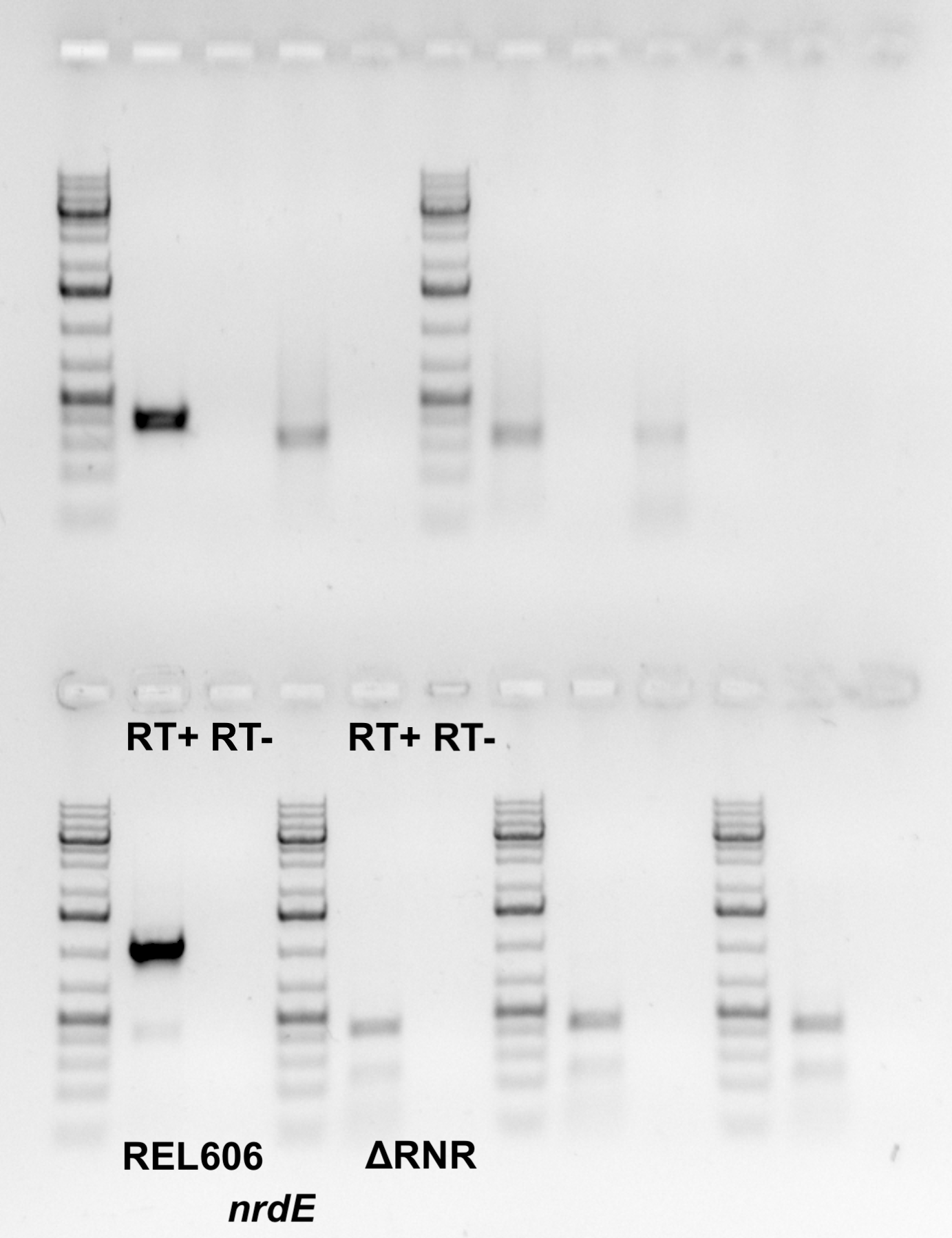

Supplement: Figure 1—figure supplement 1—source data 1. [file elife-83845-fig1-figsupp1-data1.zip › Figure_1-figure_supplement1_source_data/Figure_1-figure_supplement_source_data_10.tif]

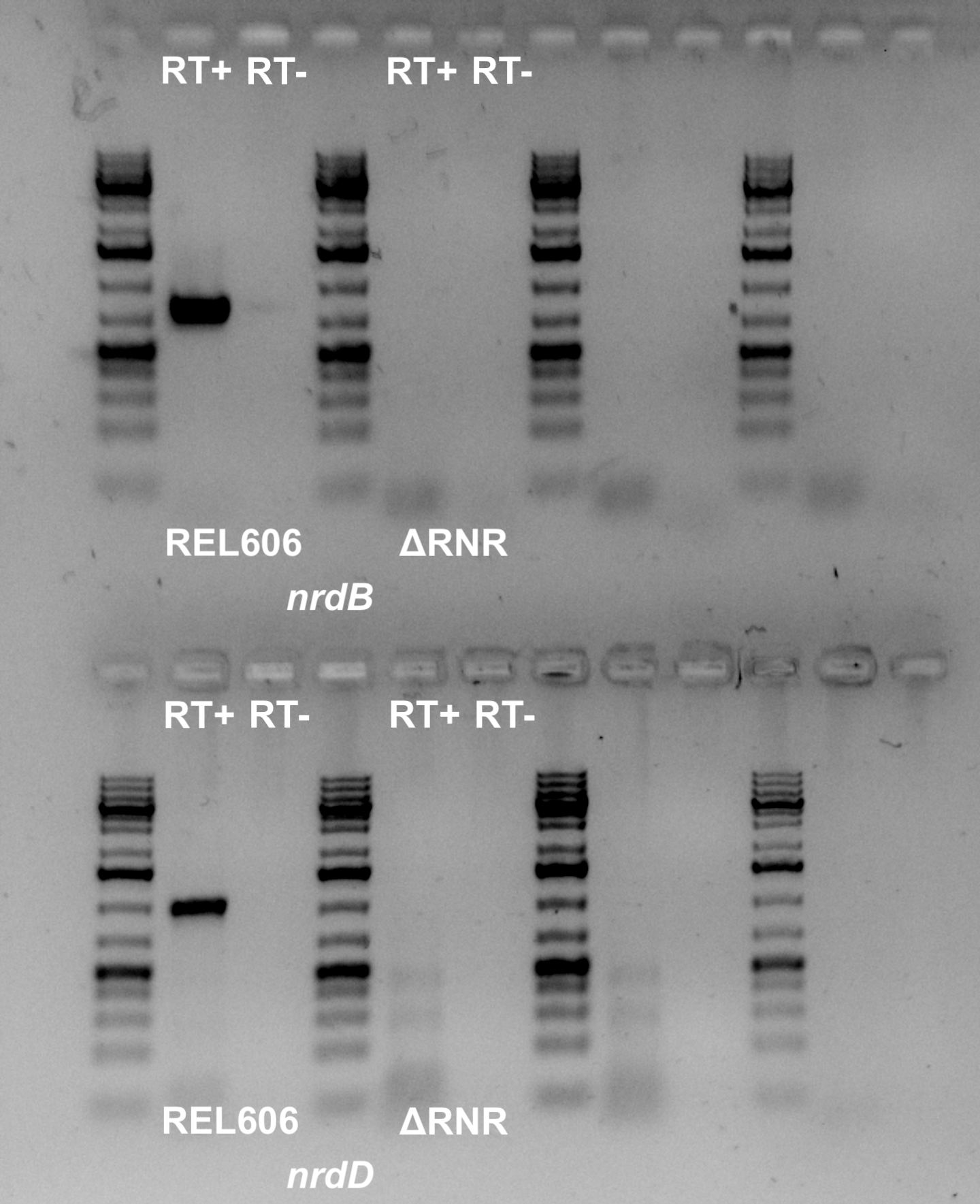

Supplement: Figure 1—figure supplement 1—source data 1. [file elife-83845-fig1-figsupp1-data1.zip › Figure_1-figure_supplement1_source_data/Figure_1-figure_supplement_source_data_8.tif]

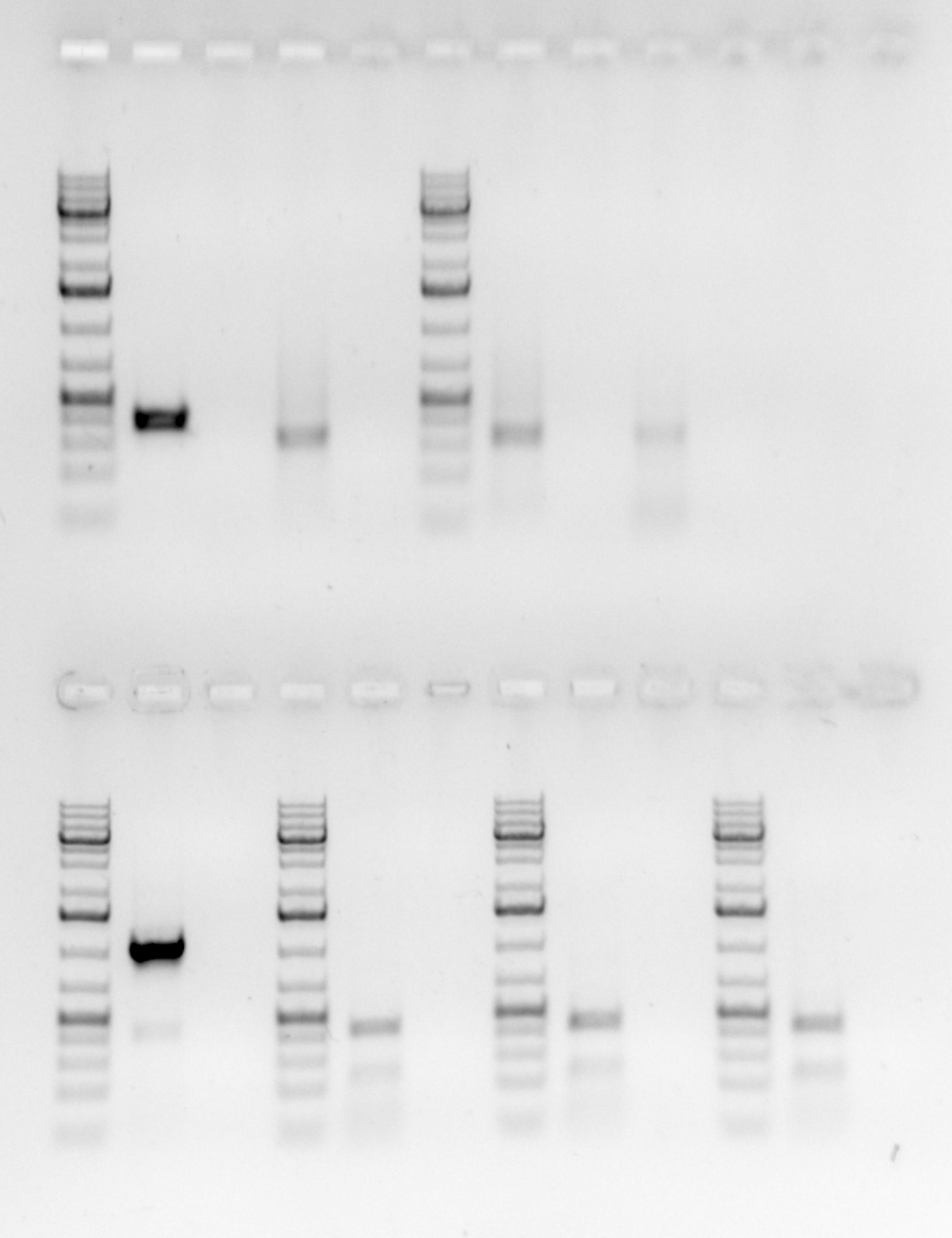

Supplement: Figure 1—figure supplement 1—source data 1. [file elife-83845-fig1-figsupp1-data1.zip › Figure_1-figure_supplement1_source_data/Figure_1-figure_supplement_source_data_9.tif]

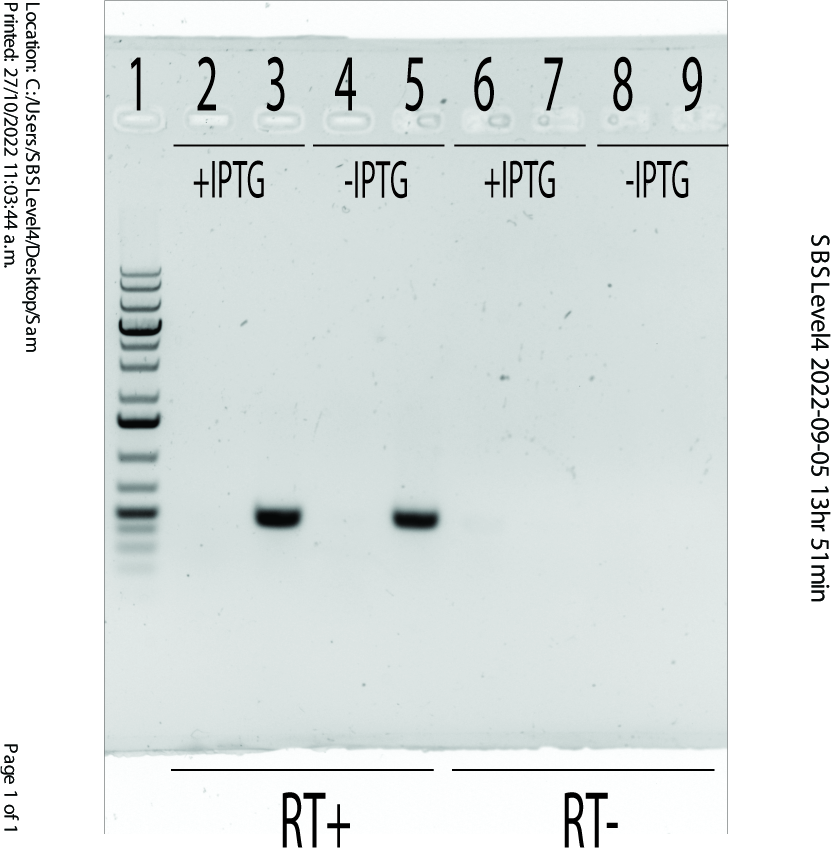

Supplement: Figure 1—figure supplement 2—source data 1. [file elife-83845-fig1-figsupp2-data1.zip › Figure_1-figure_supplement2_source_data/Figure_1-figure_supplement2_source_data_2.tif]

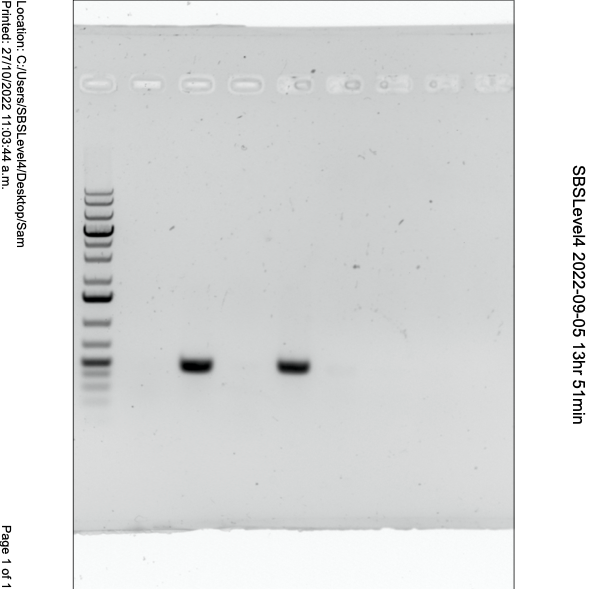

Supplement: Figure 1—figure supplement 2—source data 1. [file elife-83845-fig1-figsupp2-data1.zip › Figure_1-figure_supplement2_source_data/Figure_1-figure_supplement2_source_data_1.tif]

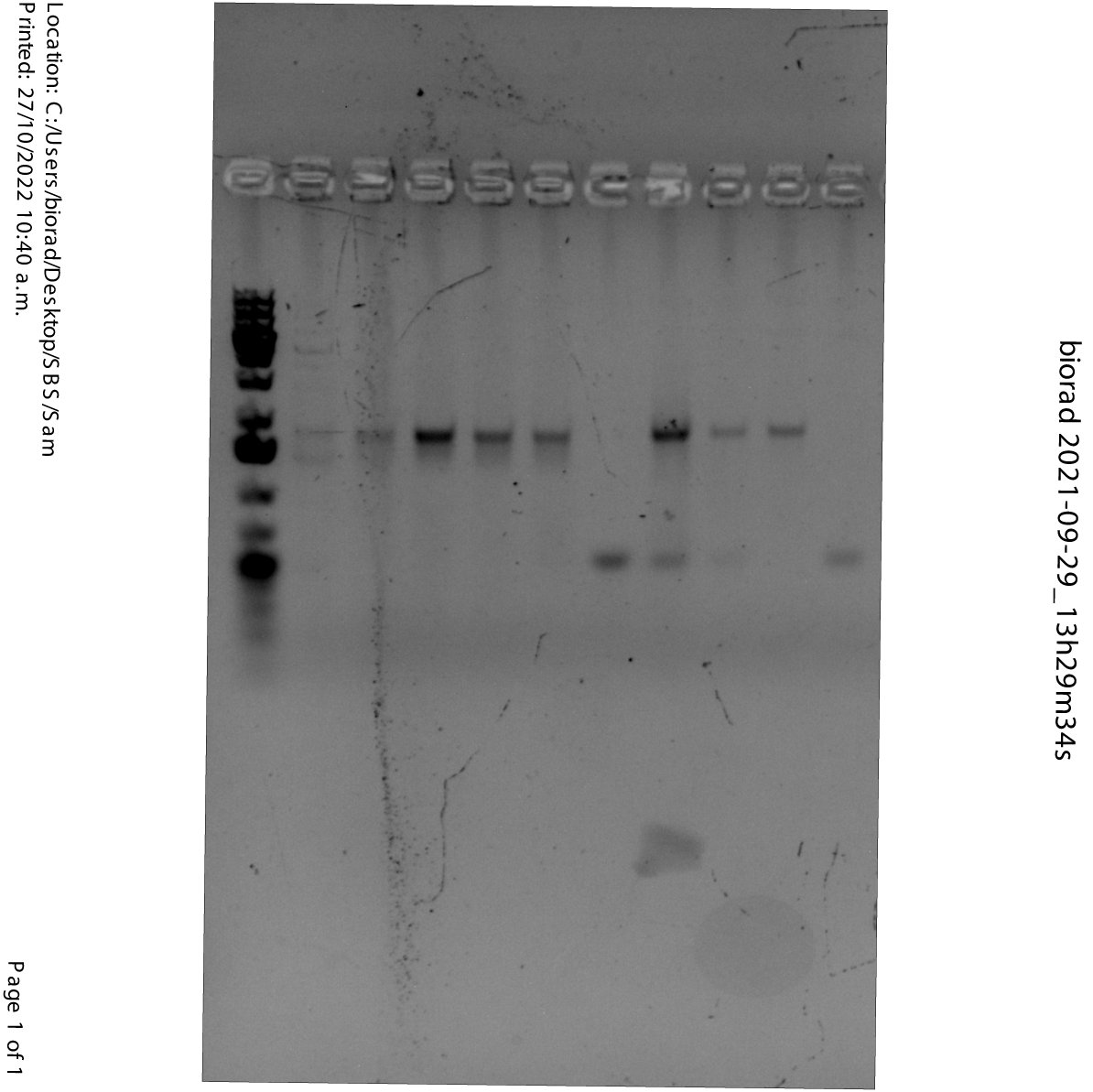

Supplement: Figure 8—figure supplement 3—source data 1. [file elife-83845-fig8-figsupp3-data1.zip › Figure_8-figure_supplement3_source_data/Figure_8-figure_supplement3_source_data_7.tif]

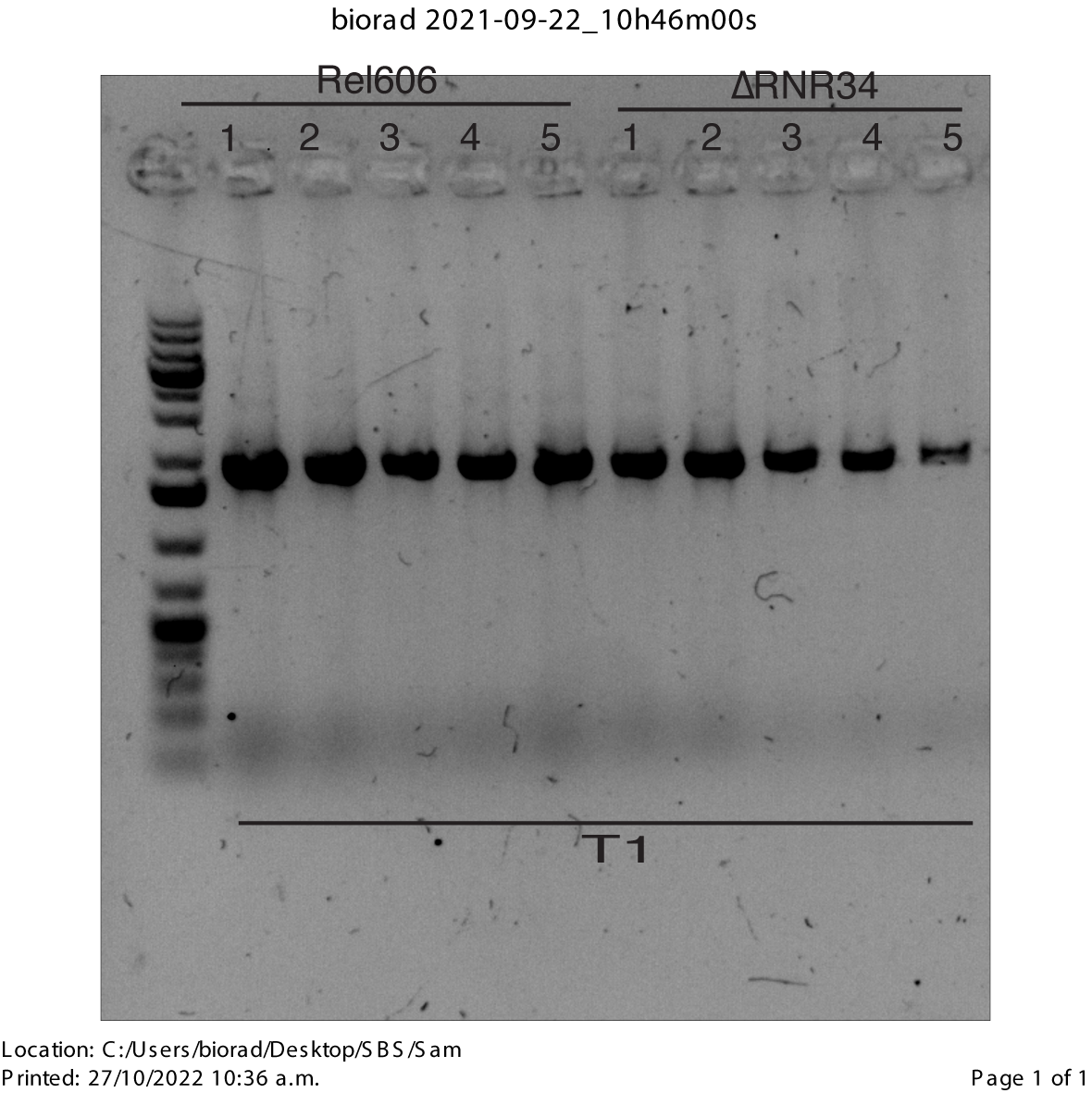

Supplement: Figure 8—figure supplement 3—source data 1. [file elife-83845-fig8-figsupp3-data1.zip › Figure_8-figure_supplement3_source_data/Figure_8-figure_supplement3_source_data_6.tif]

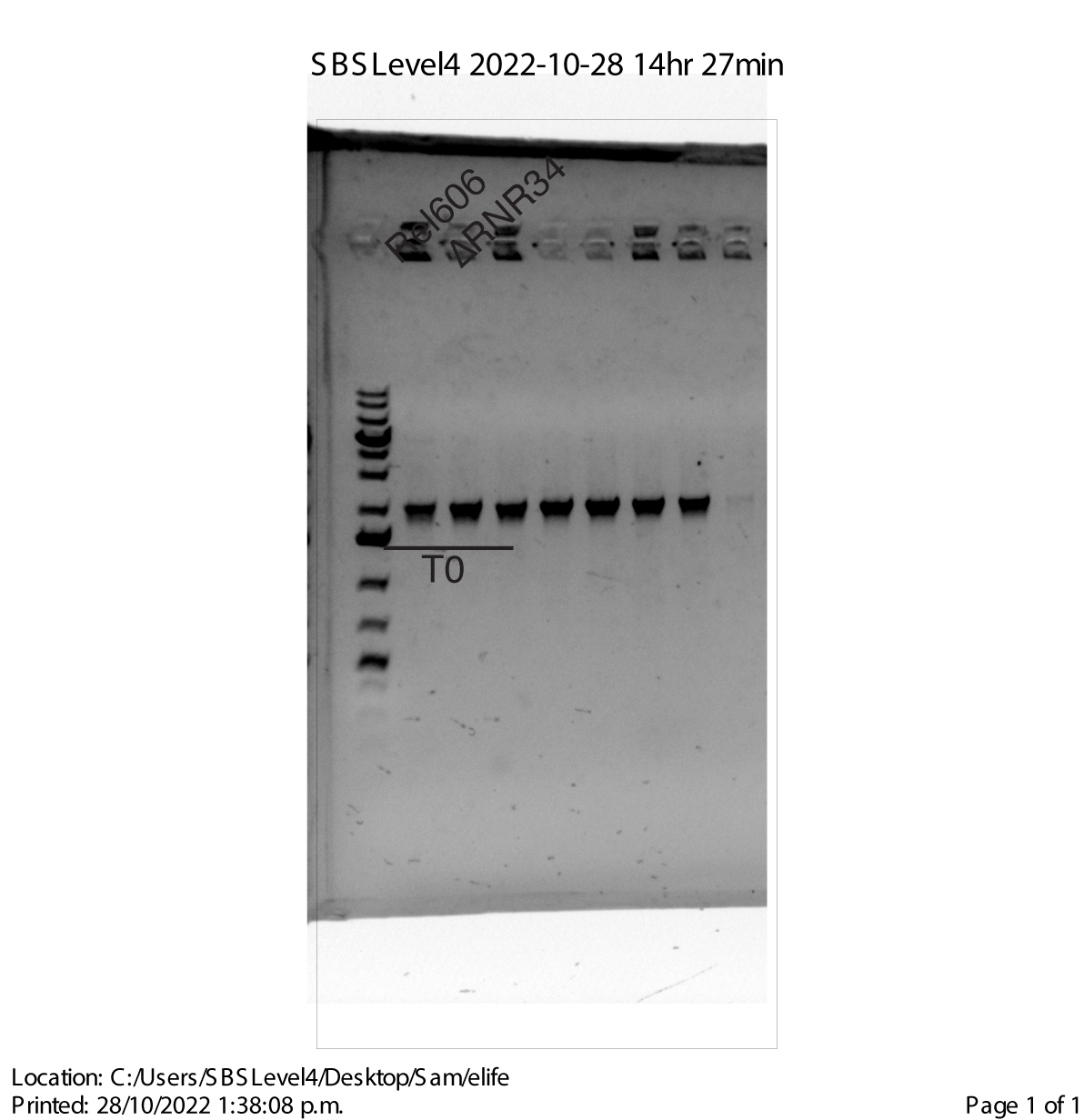

Supplement: Figure 8—figure supplement 3—source data 1. [file elife-83845-fig8-figsupp3-data1.zip › Figure_8-figure_supplement3_source_data/Figure_8-figure_supplement3_source_data_4.tif]

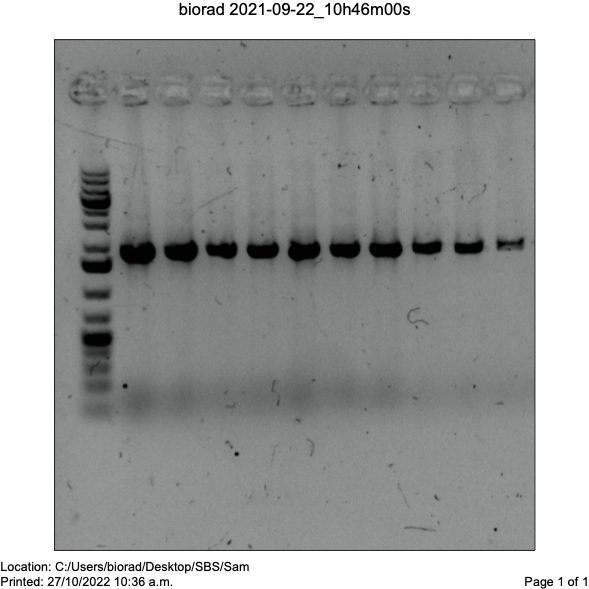

Supplement: Figure 8—figure supplement 3—source data 1. [file elife-83845-fig8-figsupp3-data1.zip › Figure_8-figure_supplement3_source_data/Figure_8-figure_supplement3_source_data_5.tif]

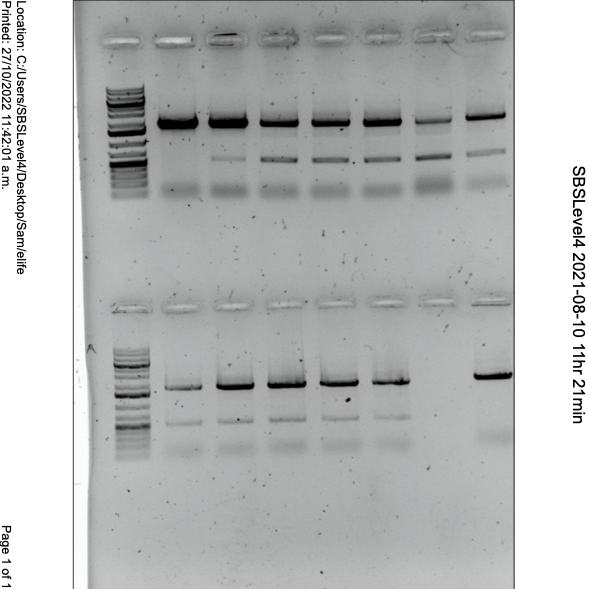

Supplement: Figure 8—figure supplement 3—source data 1. [file elife-83845-fig8-figsupp3-data1.zip › Figure_8-figure_supplement3_source_data/Figure_8-figure_supplement3_source_data_1.tif]

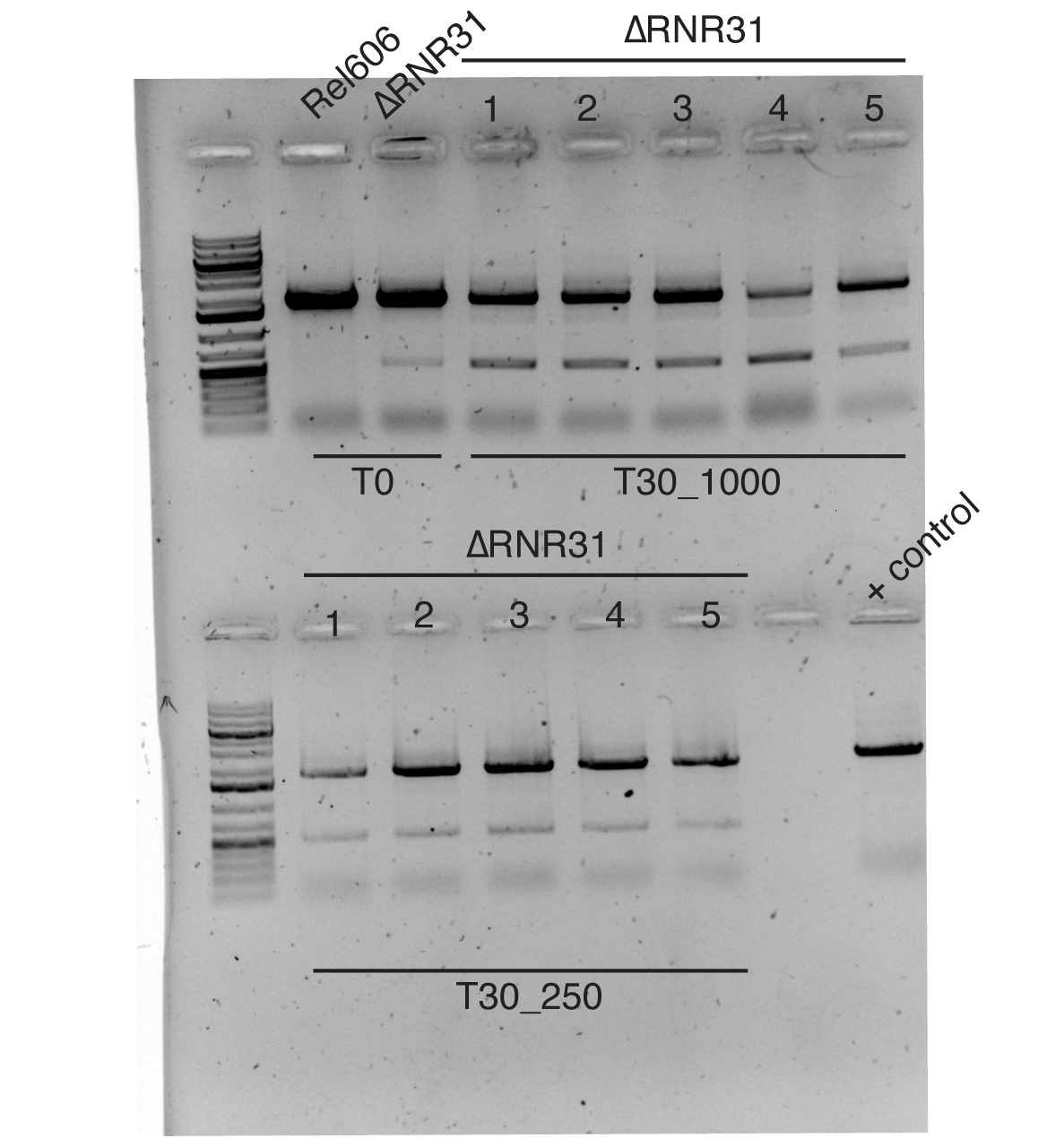

Supplement: Figure 8—figure supplement 3—source data 1. [file elife-83845-fig8-figsupp3-data1.zip › Figure_8-figure_supplement3_source_data/Figure_8-figure_supplement3_source_data_2.tif]

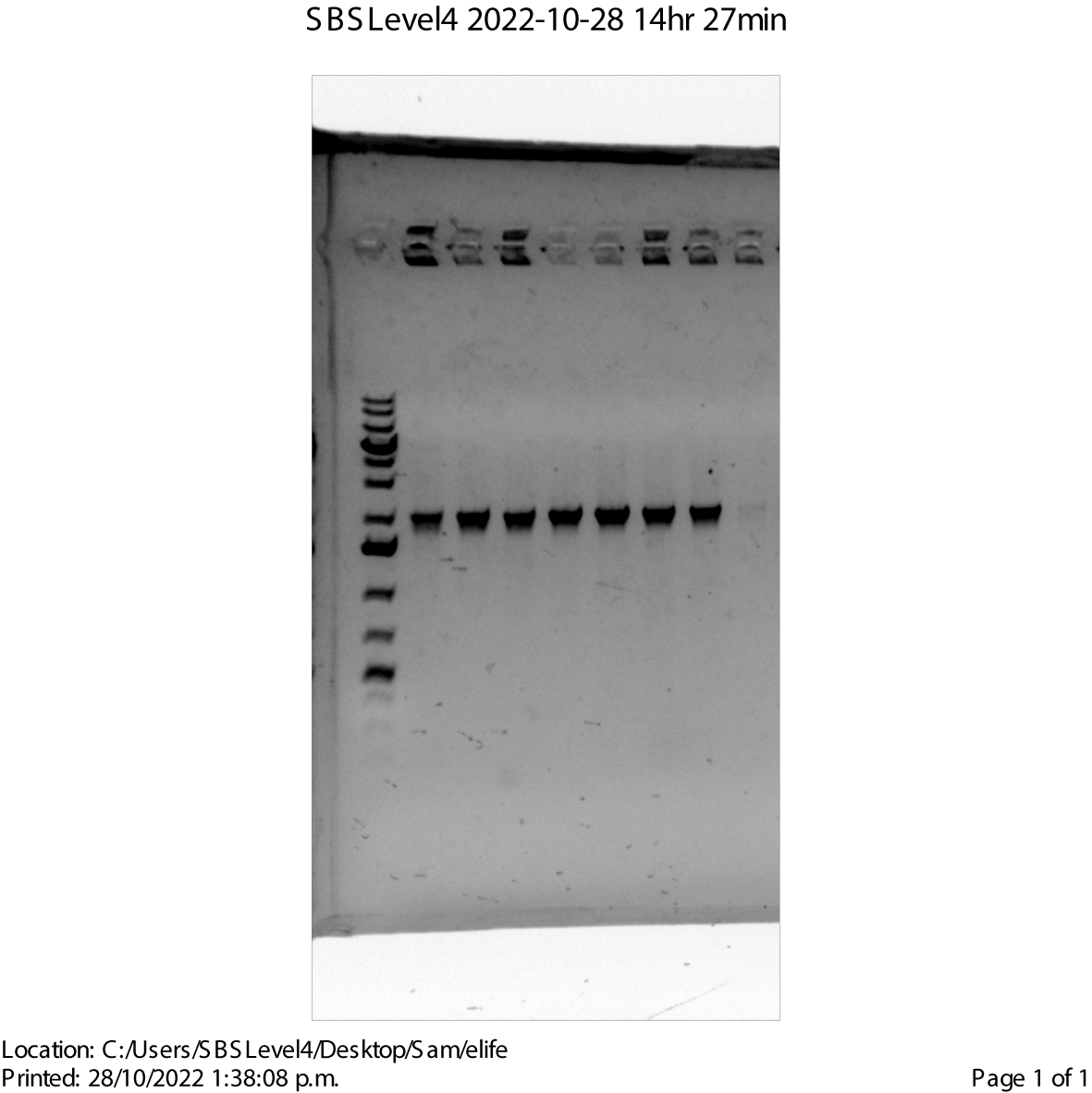

Supplement: Figure 8—figure supplement 3—source data 1. [file elife-83845-fig8-figsupp3-data1.zip › Figure_8-figure_supplement3_source_data/Figure_8-figure_supplement3_source_data_3.tif]

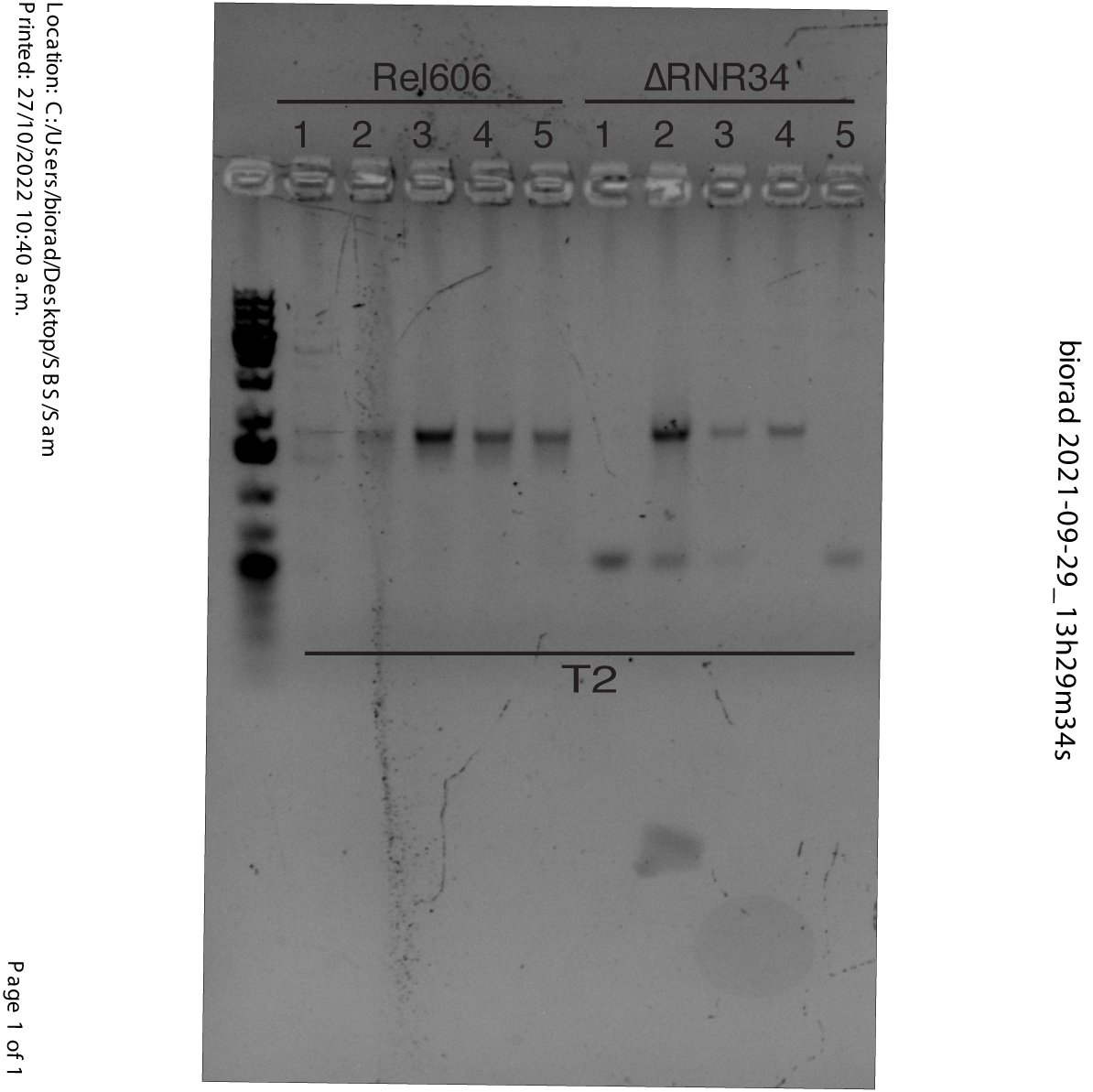

Supplement: Figure 8—figure supplement 3—source data 1. [file elife-83845-fig8-figsupp3-data1.zip › Figure_8-figure_supplement3_source_data/Figure_8-figure_supplement3_source_data_8.tif]
