# Supplementary material for: Characterisation of an Escherichia coli line that completely lacks ribonucleotide reduction yields insights into the evolution of parasitism and endosymbiosis
Source: eLife. 2023 Apr 6;12:e83845. doi: 10.7554/eLife.83845 (PMC10121223; doi:10.7554/eLife.83845)
Supplement: Supplementary file 3. [file elife-83845-supp3.zip › Supplementary_File3.rtf]

Supplementary File 3: Alignment of phosphopentomutases.Consensus                   MNMMKRAFIMVLDSFGIGAAADADXF----------GDVGADTLGHIAEACAEGEADN-G-RKGPLKLPNLSRLGLGKAA   68deoB_Bcereus_WP_098782964.1 MNKYKRIFLVVMDSVGIGEAPDAEQF----------GDLGSDTIGHIAE------------HMNGLQMPNMVKLGLGNIR   58deoB_Rel606                 ---MKRAFIMVLDSFGIGATEDAERF----------GDVGADTLGHIAEACAKGEADN-G-RKGPLNLPNLTRLGLAKAH   65sp|Q89A57|DEOB_BUCBP        ---MKRAFLIVLDSFGIGATKDAHRF----------GDVGANTFGNIAKFCFLNKANNYG-RKGLLYIPHLLSLGLAKVA   66sp|Q8K936|DEOB_BUCAP        ---MKRVFLMILDSLGIGSSVDACKF----------NDSGADTLGHIAEKCFFNEANVQ--RRGSLYIPNLIKLGIVKSY   65sp|B8D866|DEOB_BUCAT        ---MKRVFLIVLDSFGIGSSPDADKF----------NDVGSNTFGHIVEKCFLGEANV-G-RKGVLCIPNLVKLGIINAA   65sp|P57607|DEOB_BUCAI        ---MKRVFLIVLDSFGIGSSPDADKF----------NDVGSNTFGHIVEKCFLGEANV-G-RKGVLCIPNLVKLGIINAA   65sp|B8D9W4|DEOB_BUCA5        ---MKRVFLIVLDSFGIGSSPDADKF----------NDVGSNTFGHIVEKCFLGEANV-G-RKGVLCIPNLVKLGIINAA   65sp|Q483R0|DEOB_COLP3        ---MARAIIIVIDSLGIGYSPDAVDF----------GDVGANTFANLARAYYEETGKE-------IFLPNLSALGMIKAC   60sp|Q3ICU9|DEOB_PSET1        ---MARAIILMADSLGIGAAPDADKF----------GDIGANTLAHLLKAYYDETGTA-------LSLPNLSKLGLIDAC   60sp|Q5QXT9|DEOB_IDILO        ---MSRAIVLVLDSFGIGSAPDADKF----------GDEGADTFGHIAEYRYQ--------HGQPLQLPNLTRLGLAEAH   59sp|Q17YP1|DEOB_HELAH        --MQKRVVVLLLDSFGIGASEDAKDF----------GDLGANTLGNIAKACFNNLADSND-RNGALKLPNLESLGLGLSA   67sp|Q9ZK37|DEOB_HELPJ        --MQKRVVILLLDSFGIGASEDAKDF----------GDLGANTLGNIAKACFNNLADSND-RNGVLKLPNLEGLGLGLSA   67sp|B6JN18|DEOB_HELP2        --MQKRVVILLLDSFGIGASEDAKDF----------GDLGANTLGNIAKACFNNLADSND-RSGALKLPYLEGLGLGLSA   67sp|P56195|DEOB_HELPY        --MQKRVVILLLDSFGIGASEDAKDF----------GDLGANTLGNIAKACFNNLADSND-RSGALKLPYLESLGLGLSA   67sp|B5Z8H3|DEOB_HELPG        --MQKRVVVLLLDSFGIGASEDAKDF----------GDFGANTLGNIAKACFNNLANSND-RNGALKLPYLESLGLGLSA   67sp|B2UUU2|DEOB_HELPS        --MQKRVVILLLDSFGIGASEDARDF----------GDLGANTLGNIAKACFNNLADSND-RNGALKLPYLESLGLGLSA   67sp|Q1CS87|DEOB_HELPH        --MQKRVVILLLDSFGIGASEDAKDF----------GDLGANTLGNIAKACFNNLADSND-RKGALKLPYLESLGLGLSA   67sp|A4YPV1|DEOB_BRASO        ----MRALIVVMDSVGIGGAPDAAGY----------GDEGADTVGHIAEACAAGQADSDQ-RQGPLQLPHLAALGLGEAC   65sp|B8IJW7|DEOB_METNO        ---MSRALLIVLDSVGIGGAPDAASY----------GDEGADTVGHIAASCARGAGDRAGLRQGPLRLPHLAALGLGLAA   67sp|B0UPC1|DEOB_METS4        ---MTRALVIVLDSVGIGGAPDGAAY----------GDGGADTLGHIAEACAEGRGDRPGLRAGPLRLPHLAGLGLGLAA   67sp|B1LYJ9|DEOB_METRJ        ---MARALIIVLDSVGVGGAPDADRY----------GDTGSDTLGHIAERCAEGRGDRAGLRAGPLRLPHLAELGLGLAA   67sp|B1ZIG8|DEOB_METPB        ---MARALLIVLDSVGIGGAPDADRY----------GDAGSDTVGHIAEACAAGRGDRPGLRAGPLRLPNLAALGLGLAC   67sp|A9W427|DEOB_METEP        ---MARALLIVLDSVGIGGAPDADRY----------GDAGSDTVGHIAEACAAGRGDRPGLRAGPLHLPNLAALGLGLAC   67sp|B7KXI3|DEOB_METC4        ---MARALLIVLDSVGIGGAPDADRY----------GDAGSDTVGHIAEACAAGRGDRPGLRAGPLRMPNLTALGLGLAC   67sp|C3MBH5|DEOB_SINFN        ---MARVFLFVLDSFGIGGAPDAEAF----------GDLGADTLGHIAEFCASGAGDRAGLREGPLQLPNMSALGLVHAA   67sp|Q92T47|DEOB_RHIME        ---MARAFLFVLDSFGIGNAPDAEAF----------GDLGADTLGHIAEFCAAGAADRAGLREGPLHLPNMSALGLMHAA   67sp|A6UET6|DEOB_SINMW        ---MARAFLFVLDSFGIGNAPDAGAF----------GDLGADTLGHIAEFCAAGAADRAGLREGPLNLPNMSALGLMHAA   67sp|B9JYP7|DEOB_AGRVS        ---MARAFLFVLDSFGVGGAPDAPAY----------GDDGADTLGHIAEFCAAGAGDRDGLRAGPLMLPNLSALGLLQIA   67sp|Q8UJ04|DEOB_AGRFC        ---MARAFLLVLDSFGVGGAPDAERY----------GDLGANTLGHIAEFCAAGAADRAGLRAGPLKLPNMCSLGLLEIA   67sp|B9J6V9|DEOB_AGRRK        ---MARAFLFVLDSFGVGGSPDATSY----------GDEGADTLGHIAEFCAAGAADREGLRSGPLSLPIMSGLGLLEIA   67sp|Q2KDR4|DEOB_RHIEC        ---MARAFLFVLDSFGVGGAPDAAAY----------GDEGADTLGHIAEFCAAGAGDRAGLRSGPLSLPNMSELGLMHIA   67sp|B5ZXI4|DEOB_RHILW        ---MARAFLFVLDSFGVGGAPDAAAY----------GDEGADTLGHIAEFCAAGAGDRAGLRDGPLSLPNLSELGLMQIA   67sp|Q1MMV6|DEOB_RHIL3        ---MARAFLFVLDSFGVGGAPDAAAY----------GDEGADTLGHIAEFCAAGAADRAGLREGPLSLPNMSELGLMQIA   67sp|Q98BG5|DEOB_RHILO        ---MARAFLFVLDSFGIGGAADAERY----------GDAGANTLAHIAEACAEGRADRERLRQGPLFVPHMASLGLGKAA   67sp|Q11AV9|DEOB_CHESB        ---MARAFLFVLDSFGIGHAPDAARF----------GDEGANTFGHIAGACAAGRADRAGLRTGPLDLPHMQSLGLNAAA   67sp|A5IGR8|DEOB_LEGPC        --MTGRVCVLVMDSFGIGASLDAARY----------GDVGANTLVHIYEACKRGECDIDGVRKGSLMLPNLASKGLYHAA   68sp|Q5X7B2|DEOB_LEGPA        --MTGRVCVLVMDSFGIGASLDAARY----------GDAGANTLVHIYEACKRGECDIDGVRKGPLMLPNLAGKGLYHAA   68sp|Q5WYR0|DEOB_LEGPL        --MTGRICILVMDSFGIGASLDAARY----------GDAGANTLVHIYEACKRGECDIEGARKGPLMLPNLAGKGLYHAA   68sp|Q5ZXU2|DEOB_LEGPH        --MTGRVCVLVMDSFGIGASLDAARY----------GDAGANTLVHIYEACKRGECDIEGVRKGPLMLPNLAGKGLYHAA   68sp|B2KBT7|DEOB_ELUMP        ---MKRVIILMMDSFGIGGAADAAKF----------GDEGANTLASVAK------------LNNGLKIPNLISLGLVKAA   55sp|Q2SHN3|DEOB_HAHCH        ---MSRAILIVLDSFGIGATADAQRF----------GDAGANTLLHIAEACAAGACDSQG-RTGPLRLPNLNRLGLGRAG   66sp|Q1QZC2|DEOB_CHRSD        ---MTRAILLVLDSFGIGNAPDAAAF----------GDAGADTLGHIAHHRAAS-----G-R--PLALPHLASLGLYHAH   59sp|A1SYK3|DEOB_PSYIN        ---MKRTVILLLDSFGVGGAADADKFIGSLADGSQYDDMGSNTLGHIAEQCAKGNANE-G-RHGPLTIPNLNKLGFGRAC   75sp|A6VM02|DEOB_ACTSZ        ---MKRVLIMMLDSFGIGGAEDADKF----------GDKGANTLGHIAS------------HQPSLNLPHLESLGLGLAA   55sp|C4LAY9|DEOB_TOLAT        ---MKRTIILMLDSLGIGASADAVRF----------GDEGANTLGHIAQACVTGNADN-G-RKGPLTLPNLTRLGLAHAC   65sp|A4SRU2|DEOB_AERS4        ---MKRTFILMMDSFGIGAAADADKF----------GDVGANTLGHIAKACAAGDIE----GRSALNLPNLNKLGLGHAG   63sp|A0KPE2|DEOB_AERHH        ---MKRTFILMMDSFGIGAAADADKF----------GDVGANTLGHIAKACAAGEIE----GRGALNLPNLSKLGLGHAG   63sp|Q7NRT1|DEOB_CHRVO        ---MKRAIILILDSLGIGAAADAPKF----------GDAGSNTLGHIAMEAAAGRADI-G-RSGPLKLPNLSRMGLGHAC   65sp|Q086F8|DEOB_SHEFN        ---MKRTFILMLDSFGVGAATDADKF----------GDVGSDTFGHIAQACADGKADI-G-RQGPLKLPNLARFGLGHAG   65sp|Q12QG1|DEOB_SHEDO        ---MKRTFILMLDSFGIGAASDADKF----------GDIGADTFGNIAKACAEGKANI-G-RDGSLKLPNLAKLGLGHAG   65sp|A1S476|DEOB_SHEAM        ---MKRTIILMLDSFGIGAADDADKF----------GDVGSDTFGHIAQMCAEGKANN-G-REGELKLPNLSRLGLAHAA   65sp|Q8EHK2|DEOB_SHEON        ---MKRTVIMMLDSFGVGAAGDAAKF----------GDVGSDTFGHIAKACAEGKADT-G-RKGPLALPNLARLGLAHAA   65sp|A0KU09|DEOB_SHESA        ---MKRTVIMMLDSFGVGAAGDAAKF----------GDLGSDTFGHIAKACAEGEADI-G-REGPLTLPNLARLGLAHAA   65sp|Q0HLE8|DEOB_SHESM        ---MKRTVIMMLDSFGVGAAGDAAKF----------GDLGSDTFGHIAKACAEGKADI-G-REGPLTLPNLARLGLAHAA   65sp|Q0HXQ2|DEOB_SHESR        ---MKRTVIMMLDSFGVGAAGDAAKF----------GDLGSDTFGHIAKACAEGKADI-G-REGPLTLPNLARLGLAHAA   65sp|A1RH89|DEOB_SHESW        ---MKRTIIMMLDSFGVGASADAASF----------GDVGSDTFGHIAKACAEGKADI-G-REGPLKLPNLARLGLGHAA   65sp|A4Y9A6|DEOB_SHEPC        ---MKRTIIMMLDSFGVGASADAASF----------GDVGSDTFGHIAKACAEGKADI-G-REGPLKLPNLARLGLGHAA   65sp|A6WRB6|DEOB_SHEB8        ---MKRTIIMMLDSFGVGASADAASF----------GDVGSDTFGHIAKACAEGKADI-G-REGPLKLPNLARLGLGHAA   65sp|A9KZ78|DEOB_SHEB9 (2)    ---MKRTIIMMLDSFGVGASADAASF----------GDVGSDTFGHIAKACAEGKADI-G-REGPLKLPNLARLGLGHAA   65sp|B8E6P6|DEOB_SHEB2        ---MKRTIIMMLDSFGVGASADAASF----------GDVGSDTFGHIAKACAEGKADI-G-REGPLKLPNLARLGLGHAA   65sp|B0TQ89|DEOB_SHEHH        ---MKRTIIMMLDSFGIGEAHDAHAF----------GDVGSNTFGNIAKACAEGKAND-G-REGPLKLPNLARLGLGHAS   65sp|A8H726|DEOB_SHEPA        ---MKRTIIMMLDSFGVGAAHDAEAF----------GDVGSNTFGHIAKACAEGKAND-G-REGPLKLPNLARLGLGHAS   65sp|A8FYQ7|DEOB_SHESH        ---MKRTFIMMLDSFGIGAAADAEAF----------GDTGSDTFGNIAKACAEGKANE-G-REGPLKLPNLARLGLAQAS   65sp|A3QGT1|DEOB_SHELP        ---MKRTVIMMLDSFGVGAAKDAEAF----------GDTGSNTFGHIAKACAEGKAND-G-REGPLKLPNLAKLGLALAA   65sp|B1KRP6|DEOB_SHEWM        ---MKRTIIMMLDSFGVGAATDAESF----------GDVGSDTFGSIAKACAEGRADI-G-REGPLKLPNLSKLGLALAA   65sp|Q6LUH2|DEOB_PHOPR        ---MKRSIILVLDSFGIGATEDAVDF----------GDVGSNTMGHIAQACARGEADN-GDRSGPLHLPNLNKLGLGKAC   66sp|B6ENG5|DEOB_ALISL        ---MKRAIILVLDSFGIGAAGDAEKF----------GDVGSDTMGHIAEQCDKGLADN-GNRKGPLTLPNLSKLGLAMAG   66sp|B5FAA0|DEOB_ALIFM        ---MKRAIILVLDSFGIGAAGDADKF----------GDVGSDTMGHIAEQCDKGLADN-GNRKGSLTLPNLSKLGLAMAG   66sp|Q5E7J5|DEOB_ALIF1        ---MKRAIILVLDSFGIGAAGDADKF----------GDVGSDTMGHIAEQCDKGLADN-GNRKGPLTLPNLSKLGLAMAG   66sp|C3LQC0|DEOB_VIBCM (2)    ---MKRAFILVLDSFGIGATADAQAF----------GDVGSDTLGHIADQCAQGLADNAE-RKGALQLPNLSKLGLAMAH   66sp|Q9KPL9|DEOB_VIBCH        ---MKRAFILVLDSFGIGATADAQAF----------GDVGSDTLGHIADQCAQGLADNAE-RKGALQLPNLSKLGLAMAH   66sp|A7MUW4|DEOB_VIBCB        ---MKRAFILVLDSFGIGAAADAEQF----------GDVGSDTLGHIADQCEQGLANNDK-REGALRLPNLSKLGLAMAH   66sp|Q7MI40|DEOB_VIBVY (2)    ---MKRAFILVLDSFGIGATADAKEF----------GDVGSDTLGHIADQCEQGLANNDQ-RQGALRLPNLSKLGLAMAH   66sp|Q87M24|DEOB_VIBPA        ---MKRAFILVLDSFGIGATADAKEF----------GDVGSDTLGHIADQCEKGLADNDK-RQGALRLPNLSKLGLAMAH   66sp|Q2NW04|DEOB_SODGM        ---MQRVFIMVLDSFGIGAAEDADKF----------GDRGSDTLGHIAERCERGDANH-G-RKGPLKLPNLTRLGLAKAA   65sp|B2VH52|DEOB_ERWT9        ---MKRAFIMVLDSFGIGSSKDAEKF----------GDKGSDTLGHIAEACFRGEADK-G-RKGPLHLPNLTALGLGKAA   65sp|A7MGA8|DEOB_CROS8        ---MKRAFIMVLDSFGIGATEDAERF----------GDAGSDTLGHIAEACAKGEADI-G-RKGPLHLPNLTKLGLAKAH   65sp|A4W6A0|DEOB_ENT38        ---MKRAFIMVLDSFGIGATEDADRF----------GDVGSDTMGHIAEACAKGEADI-G-RQGPLNLPNLTRLGLVKAH   65sp|Q327L3|DEOB_SHIDS        ---MKRAFIMVLDSFGIGATEDAERF----------GDVGADTLGHIAEACAKGKADN-G-RKGPLNLPNLTRLGLAKAH   65sp|Q1R260|DEOB_ECOUT (3)    ---MKRAFIMVLDSFGIGATEDAERF----------GDVGADTLGHIAEACAKGEADH-G-RKGPLNLPNLTRLGLAKAH   65sp|B7UR11|DEOB_ECO27        ---MKRAFIMVLDSFGIGATEDAERF----------GDVGADTLGHIAEACAKGEADN-G-RKGPLNLPNLTRLGLAKAH   65sp|Q3YU10|DEOB_SHISS        ---MKRAFIMVLDSFGIGATEDAERF----------GDVGADTLGHIAEACAKGEADN-G-RKGPLNLPNLTRLGLAKAH   65sp|Q0SX28|DEOB_SHIF8 (22)   ---MKRAFIMVLDSFGIGATEDAERF----------GDVGADTLGHIAEACAKGEADN-G-RKGPLNLPNLTRLGLAKAH   65sp|B5Y275|DEOB_KLEP3        ---MKRAFIMVLDSFGIGATEDADRF----------GDVGADTMGHIAEACAKGEADN-G-RKGPLNLPNLTRLGLVKAH   65sp|A9MRA5|DEOB_SALAR        ---MKRAFIMVLDSFGIGATEDADRF----------GDVGSDTLGHIAEACAKGEADN-G-RKGPLNLPNLTRLGLVKAH   65sp|B5BAJ9|DEOB_SALPK (2)    ---MKRAFIMVLDSFGIGATEDADRF----------GDVGSDTLGHIAEACAKGEADN-G-RKGPLNLPNLTRLGLVKAY   65sp|C0Q7M5|DEOB_SALPC (2)    ---MKRAFIMVLDSFGIGATEDADRF----------GDVGSDTLGHIAEACANGEADN-G-RKGPLNLPNLTRLGLVKAH   65sp|B5R9V1|DEOB_SALG2        ---MKRAFIMVLDSFGIGATADADRF----------GDVGSDTLGHIAEACAKGEADN-G-RKGPLNLPNLTRLGLVKAH   65sp|B5FTC7|DEOB_SALDC        ---MKRAFIMVLDSFGIGATEDADRF----------GDVGSDTLGHIAEACAKGEADN-G-RKGPLNLPNLTRLGLVKAH   65sp|P63924|DEOB_SALTI (8)    ---MKRAFIMVLDSFGIGATEDADRF----------GDVGSDTLGHIAEACAKGEADN-G-RKGPLNLPNLTRLGLVKAH   65sp|C5BHJ4|DEOB_EDWI9        ---MKRAFIMVLDSFGIGEAKDAKSF----------GDEGADTLGHIARACARGEADI-G-RQGPLHLPNLSRLGLGKAA   65sp|Q6D990|DEOB_PECAS        ---MKRVYIMVLDSFGIGSSADAERF----------GDVGSDTLGHIAQACAAGTADK-G-RSGSLHLPNLSRLGLGKAA   65sp|C6DKL9|DEOB_PECCP        ---MKRAYIMVLDSFGIGSSADAERF----------GDAGSDTLGHIAQACAAGTADK-G-RSGPLHLPNLSRLGLGKAA   65sp|B4EWA2|DEOB_PROMH        ---MKRVHIMVLDSFGIGAAGDAEKF----------GDQGADTLGHIAQAFAEGKADKDG-RKGPLHLPNLCRLGLGKAA   66sp|Q7N931|DEOB_PHOLL        ---MKRTFIMVLDSFGIGASKDAEKF----------GDQGSNTLGHIAEACARGDADI-G-RKGPLYLPNLSRLGLGKAT   65sp|A8G9H8|DEOB_SERP5        ---MKRTFIMVLDSFGIGASEDAAKF----------GDQGSDTLGHIAEVCARGEANV-G-RQGPLTLPNLSRLGLGKAA   65sp|A1JJ99|DEOB_YERE8        ---MKRTFIMVLDSFGIGASADANKF----------GDEGADTLGHIAEACARGEADI-G-RSGPLTLPNLSRLGLGKAA   65sp|A9R047|DEOB_YERPG        ---MKRTFIMVLDSFGIGASADAKKF----------GDEGADTLGHIAEACARGEANV-G-RSGPLTLPNLSRLGLGKAA   65sp|A4TQJ1|DEOB_YERPP (4)    ---MKRTFIMVLDSFGIGASADAKKF----------GDEGADTLGHIAEACARGEANV-G-RSGPLTLPNLSRLGLGKAA   65sp|B1JL35|DEOB_YERPY (4)    ---MKRTFIMVLDSFGIGASADAKKF----------GDEGADTLGHIAEACARGEANV-G-RSGPLTLPNLSRLGLGKAA   65Consensus                   EESTG-RFP-AGLD--DNAEVIGAY-GHASELSSGKDTPSGHWEIAGVPVLFDWGYFSDKENSFPKELLDKIVARAXLPG  143deoB_Bcereus_WP_098782964.1 EM--------KGIS--KVEKPLGYY-TKMQEKSTGKDTMTGHWEIMGLYIDTPFQVFPE---GFPKELLDELEEKTG-RK  123deoB_Rel606                 EGSTG-FIP-AGMD--GNAEVIGAY-AWAHEMSSGKDTPSGHWEIAGVPVLFEWGYFSDHENSFPQELLDKLVERANLPG  140sp|Q89A57|DEOB_BUCBP        IASSG-QDL-LGIQ--DTNNVIGSY-AYSNEISSGKDTSSGHWEIAGAPVLFDWDYFSSSLNSVPKYLIQDIVDQCNLSG  141sp|Q8K936|DEOB_BUCAP        QSSTG-KYL-LGFNQKEDANIIASY-GYASELSSGKDTTSGHWEIAGAPFLKDWYYFKEKDNSFPSSLINKIITKLKLPG  142sp|B8D866|DEOB_BUCAT        KESTG-QYP-LGFN--YSSNVIASY-GFASEISSGKDTTSGHWEIAGVPVLDDWCYFKEKQNSFPESLLEKIIRRSELTG  140sp|P57607|DEOB_BUCAI        KESTG-QYP-LGFN--YSSNVIASY-GFASEISSGKDTTSGHWEIAGVPVLDDWYYFKEKQNSFPESLLEKIIIRSELTG  140sp|B8D9W4|DEOB_BUCA5        KESTG-QYP-LGFN--YSSNVIASY-GFASEISSGKDTTSGHWEIAGVPVLDDWYYFKEKQNSFPESLLEKIIRRSELTG  140sp|Q483R0|DEOB_COLP3        EQASNQTFP---YQ--GQEPSKGAY-GFAQEISTGKDTPSGHWEMAGVPVLFDWGYFTDKNSSFPTSLIDDINRETGFDG  134sp|Q3ICU9|DEOB_PSET1        E-AAG-KEP-CLVS--NRSASQGAW-GYAKELSSGKDTPSGHWEMAGVPVLFDWGYFPKTQPSFPQEFIDELIARTGIPG  134sp|Q5QXT9|DEOB_IDILO        KEATG-TYA-KGFH---EVETHGAY-ACAAELSSGKDTPSGHWELMGVPVHFEWGYFQDKENSFPNELLAQILKACELDG  133sp|Q17YP1|DEOB_HELAH        LKATN-ELP-LGFD--SHPNLIGAY-AYAQELSSAKDTISGHWEMMGAPILFEWGYFKDKNNSFPKEILDEIMHKTKIKG  142sp|Q9ZK37|DEOB_HELPJ        LKAAN-ELP-LGFQ--LKPNLIGAY-AYAKELSSAKDTISGHWEMMGAPVLFEWGYFKDKNDSFPKEILDEIMHKTKIKG  142sp|B6JN18|DEOB_HELP2        LEAAN-ELP-LGFQ--PQPNLIGAY-AYAQELSSAKDTISGHWEMMGAPVLFEWGYFKDKNDSFPKEILDEIARKTKIKG  142sp|P56195|DEOB_HELPY        LKAAN-ELP-LGFQ--SQPNLIGAY-AYAQELSSAKDTISGHWEMMGAPVLFEWGYFKDKTHSFPKEILDEIVRKTKIKG  142sp|B5Z8H3|DEOB_HELPG        LKATN-ELP-LGFE--SKPNLIGAY-AYAKELSSAKDTISGHWEMMGAPVLFEWGYFKDKTHSFPKEILDEIMHKTKIKG  142sp|B2UUU2|DEOB_HELPS        LKATN-ELP-LGFE--SKPNLIGAY-AYAQELSSAKDTISGHWEMMGAPVLFEWGYFKDKNNSFPKEILDEIMRKTKIKG  142sp|Q1CS87|DEOB_HELPH        LKATN-ELP-LGFE--SKPNLIGAY-AYAKELSSAKDTISGHWEMMGAPVLFEWGYFKDKNNSFPKEILDEIMRKTKIKG  142sp|A4YPV1|DEOB_BRASO        EHATG-RRP-PGLQ--QKAGAGAAF-GCASERSRGKDTPSGHWEIAGVPVPFEWGYFPKTVPAFPPDLIDQLCASAGLTG  140sp|B8IJW7|DEOB_METNO        EAATG-RMP-PGLA--PAGSVTGAW-GHAVETARGKDTVSGHWEIAGVPVDFDWGRFPATEPSFPPELTQALIAEAGLPG  142sp|B0UPC1|DEOB_METS4        REASG-RMP-PGLA--PDGPVAGAW-GHAVETARGKDTVSGHWEIAGAPVDFDWGRFPATRPSFPPDLTRALIEEGNLPG  142sp|B1LYJ9|DEOB_METRJ        AGASG-RIP-PNLT--PVGPPRGVY-GHAVETAAGKDTPSGHWEIAGLPLPEPWGHFPDTRPAFPPELTRALVAEGDLPG  142sp|B1ZIG8|DEOB_METPB        EGATG-RVP-PGLA--PGEPLRALW-GHAVETAAGKDTPSGHWEIAGVPVRAPWGHFPDTHPAFPAELTAALIARAGLPG  142sp|A9W427|DEOB_METEP        EGATG-RVP-PGLA--PEGPVRALW-GHAVETAAGKDTPSGHWEIAGVPVREAWGHFPDTQPAFPAELTAALVARAGLPG  142sp|B7KXI3|DEOB_METC4        EGATG-RVP-PGLA--PDGPVRALW-GHAVETAAGKDTPSGHWEIAGVPVREAWGHFPDTQPAFPAELTAALIERAGLPG  142sp|C3MBH5|DEOB_SINFN        KLATG-RLP-AGMP--LPERVYGVY-GAASEVSRGKDTPSGHWEIAGTPVRFDWGYFPAEGDAFPPQLVEAICREGEVPG  142sp|Q92T47|DEOB_RHIME        RLATG-RLP-AGMA--LPERVYGIY-GAASEVSRGKDTPSGHWEIAGTPVTFDWGYFPAEGDAFPPELVEAICREGDVPG  142sp|A6UET6|DEOB_SINMW        RLATG-RLP-AGMA--LPERVYGVY-GAASEVSRGKDTPSGHWEIAGTPVTFDWGYFPADGDAFPPELVEAICREGDVPG  142sp|B9JYP7|DEOB_AGRVS        SLASG-SLP-AGMA--LPERVFGLY-GAANEISRGKDTPSGHWEIAGTPVMFDWGYFPQEGDAFPADLVADICKRADLPG  142sp|Q8UJ04|DEOB_AGRFC        RQASG-DIP-AGME--PPERIFGLH-GSASEISKGKDTPSGHWEIAGTPVTFDWGYFPTEGDAFSPELVEAICRQADIPG  142sp|B9J6V9|DEOB_AGRRK        KAATG-RYP-LGMP--LPQKLYGLY-GCANEISRGKDTPSGHWEIAGTPVTFDWGYFPTEGDAFPPELVEAICKAADLPG  142sp|Q2KDR4|DEOB_RHIEC        RAASG-QFP-AGMP--LPEKVYGVY-GTANEISRGKDTPSGHWEIAGTPVNFDWGYFPTEGDAFPPELIEALCRAGDVPG  142sp|B5ZXI4|DEOB_RHILW        RSASG-RFP-AGMP--IPEKVYGIY-GAATEISRGKDTPSGHWEIAGTPVSFDWGYFPTEGDAFPEELIEALCRDAGVSG  142sp|Q1MMV6|DEOB_RHIL3        RSASG-RFP-AGMP--VPEKVYGIY-GAATEISRGKDTPSGHWEIAGTPVSFDWGYFPIEGDAFPQEFIEALCREADVPG  142sp|Q98BG5|DEOB_RHILO        ETATG-----LGFAHFGTDLIANAFHGAAQEVSSGKDTPSGHWEIAGLPVRFDWGYFPDTVPAFPADLTAAMIREGEVPG  142sp|Q11AV9|DEOB_CHESB        AIAAG-REPERGFA--S----TGFF-GAAEERSTGKDTPSGHWEIAGVPVPFEWGYFPETTPSFPAELTGRLIREASLPG  139sp|A5IGR8|DEOB_LEGPC        MASSG-LPF-IDLS--ALAIPSGYY-GYAVEQSLGKDTPSGHWEMAGVPVTFEWGYFPDKPYCFPEELISEFIKQCNLPG  143sp|Q5X7B2|DEOB_LEGPA        MASSG-LPF-IDLS--ALAIPSGYY-GYAVEQSLGKDTPSGHWEMAGVPVTFEWGYFPDKSYCFPEELISEFIKQCNLPG  143sp|Q5WYR0|DEOB_LEGPL        MASSG-LPF-IDLA--TLAIPSGYY-GYAVEQSLGKDTPSGHWEMAGVPVTFEWGYFPDKPYCFPEELISEFIKQCNLPG  143sp|Q5ZXU2|DEOB_LEGPH        MASSG-LSF-IDLA--TLAIPSGYY-GYAVEQSLGKDTPSGHWEMAGVPVTFEWGYFPDKPYCFPEELISEFIKQCNLPG  143sp|B2KBT7|DEOB_ELUMP        EASAGVKIE-TGPQPPAQVNIPSKY-GFMREQSHGKDTLSGHWEMAGVPVLFDWGYFKPGYPSFPKELIEQICKEAGIDK  133sp|Q2SHN3|DEOB_HAHCH        EASCG-AAA-PGLE--QAPEIIGTY-GFAEELSSGKDTPSGHWEIAGVPVLFDWGYFTDKQQSFPPELLDAIIKEARLPG  141sp|Q1QZC2|DEOB_CHRSD        HLATG-EWA-EGIT--PPETLDGAY-AAAAEISSGKDTPSGHWEIAGVPALFEWGYFPDKTQSFPPDLLEALIEQAKLPG  134sp|A1SYK3|DEOB_PSYIN        AESSS-TFP-AGLD--PLVEPIAAY-GYAKEISTAKDTSSGHWEITGVPVLFDWGYFDKKQNSFPQELLDELVERADLPG  150sp|A6VM02|DEOB_ACTSZ        KESCG-ELP-KHFQ--NQPHLIGGY-AFAREISSGKDTTSGHWEIAGVPVLFDWGLFPDKQNSFPKPLLDRIVAKAGIKG  130sp|C4LAY9|DEOB_TOLAT        AASCG-EFP-AGLD--ASVTPIAAY-GYARELSSGKDTPSGHWEMAGAPVLFDWGYFTDKQNSFPPELLDALVAQGNLPG  140sp|A4SRU2|DEOB_AERS4        ELASG-YFP-AGLK--KDIEVVGAY-GFAQELSSGKDTPSGHWEIAGVPVLFEWGYFKDHHNSFPQELLDAIVEKAGLSG  138sp|A0KPE2|DEOB_AERHH        ELASG-TFP-AGLN--KDIDVVGAY-GFAQELSSGKDTPSGHWEIAGVPVLFEWGYFHDHHNSFPQELLDAIVEKAGLPG  138sp|Q7NRT1|DEOB_CHRVO        QLSSG-YFP-EGMD---PAAPAAAY-GYAREISSGKDTPSGHWEIAGVPVLFDWGYFSDHDNSFPQQLLDAIVDKAGLPG  139sp|Q086F8|DEOB_SHEFN        FESTG-KFA-AGFA--DNVEVIGAY-GHADELSSGKDTPSGHWEMAGVPVLYEWGYFSDLTNSFPKELTDKILERAGLDG  140sp|Q12QG1|DEOB_SHEDO        FESTG-QFA-PGFS--QDVEVVGAY-GYADELSTGKDTPSGHWEMAGVPVLYDWGYFNDLQDSFPKELTDKILARAGLSG  140sp|A1S476|DEOB_SHEAM        KEATG-AFA-PGFG--DNVDIIGAY-GHCQELSSGKDTPSGHWEMAGVPVLFEWGYFSEHQNSFPKELTDKILERAGLTE  140sp|Q8EHK2|DEOB_SHEON        MESTG-AFA-PGFA--DNVDLIGAY-GHAQELSSGKDTPSGHWEMAGVPVLFEWGYFSEHQNSFPKELTDKILARAGLDG  140sp|A0KU09|DEOB_SHESA        MESTG-AFA-PGFA--DNVELIGAY-GHAQELSSGKDTPSGHWEMAGVPVLFEWGYFSEHQNSFPKELTDKILARAGLDG  140sp|Q0HLE8|DEOB_SHESM        MESTG-AFA-PGFA--DDVELIGAY-GHAQELSSGKDTPSGHWEMAGVPVLFDWGYFSEHQNSFPKELTDKILARAGLDG  140sp|Q0HXQ2|DEOB_SHESR        MESTG-AFA-PGFA--DDVELIGAY-GHAQELSSGKDTPSGHWEMAGVPVLFDWGYFSEHQNSFPKELTDKILARAGLDG  140sp|A1RH89|DEOB_SHESW        MESTG-AFA-PGFG--DNVELIGAY-GHAQELSSGKDTPSGHWEMAGVPVLFEWGYFSEHQNSFPKELTDKILARAGLDG  140sp|A4Y9A6|DEOB_SHEPC        MESTG-AFA-PGFG--DNVELIGAY-GHAQELSSGKDTPSGHWEMAGVPVLFEWGYFSEHQNSFPKELTDKILARAGLDG  140sp|A6WRB6|DEOB_SHEB8        MESTG-AFA-PGFG--DNVELIGAY-GHAQELSSGKDTPSGHWEMAGVPVLFEWGYFSEHQNSFPKELTDKILARAGLDG  140sp|A9KZ78|DEOB_SHEB9 (2)    MESTG-AFA-PGFG--DNVELIGAY-GHAQELSSGKDTPSGHWEMAGVPVLFEWGYFSEHQNSFPKELTDKILARAGLDG  140sp|B8E6P6|DEOB_SHEB2        MESTG-AFA-PGFG--DNVELIGAY-GHAQELSSGKDTPSGHWEMAGVPVLFEWGYFSEHQNSFPKELTDKILARAGLDG  140sp|B0TQ89|DEOB_SHEHH        KESTG-EFP-AGFG--DDVEIIGAY-GHADELSSGKDTPSGHWEMAGVPVLYEWGYFGDKQNSFPKELTDKILARAGLSG  140sp|A8H726|DEOB_SHEPA        KESTG-EFP-AGFG--DDVEVIGAY-GHADELSSGKDTPSGHWEMAGVPVLYEWGYFSELTNSFPQALTDKILARAGLTE  140sp|A8FYQ7|DEOB_SHESH        KESTG-QLP-AGFS--DDVEIIGAY-GHADELSTGKDTPSGHWELAGVPVLYEWGYFSDHTNSFPKELTDKILARAGLSD  140sp|A3QGT1|DEOB_SHELP        KESTG-SFA-EGFG--DDVEVIGAY-GHADELSSGKDTPSGHWEMAGVPVLYEWGYFSDLKDSFPKELTDKILERAGLSG  140sp|B1KRP6|DEOB_SHEWM        KESTG-TFA-PGFS--DDVEVIGAY-GHADELSTGKDTPSGHWEMAGVPVLYEWGYFSDLTNSFPKELTDKILARAGLDG  140sp|Q6LUH2|DEOB_PHOPR        EESSG-YFP-EGLD--PNVEITGAY-GHAKELSSGKDTPSGHWEIAGVPVLFDWGYFSDHDNSFPKELTDRILKRANLPG  141sp|B6ENG5|DEOB_ALISL        KESTG-KLA-AGLD--ADAEIIGAY-GHAAELSSGKDTPSGHWEIAGVPVLFDWGYFSDKENSFPKELTDRILARANLSG  141sp|B5FAA0|DEOB_ALIFM        KESTG-KFS-AGLD--ANAEIIGAY-GHAAELSSGKDTPSGHWEIAGVPVLFDWGYFTDKENSFPKELTDRILERANLPG  141sp|Q5E7J5|DEOB_ALIF1        KESTG-KFS-AGLD--ANAEIIGAY-GHAAELSSGKDTPSGHWEIAGVPVLFDWGYFTDKENSFPKELTDRILERANLPG  141sp|C3LQC0|DEOB_VIBCM (2)    KESTG-RFA-PGLD--ERADIIGAY-AHAAELSSGKDTPSGHWEIAGVPVLFEWGYFSDKQNSFPKELTDRILARAGLDG  141sp|Q9KPL9|DEOB_VIBCH        KESTG-RFA-PGLD--ERADIIGAY-AHAAELSSGKDTPSGHWEIAGVPVLFEWGYFSDKQNSFPKELTDRILARAGLDG  141sp|A7MUW4|DEOB_VIBCB        KESTG-RFA-PGLD--ADAEIIGAY-GHAAELSSGKDTPSGHWEIAGVPVLFDWGYFTDKANSFPKELTDRILERAGLDG  141sp|Q7MI40|DEOB_VIBVY (2)    KESTG-RFA-PGLD--ADAEIIGAY-GHAAELSSGKDTPSGHWEIAGVPVLFEWGYFTDKANSFPKELTDRILARAGIDG  141sp|Q87M24|DEOB_VIBPA        KESTG-RFA-PGLD--ADAEIIGAY-GHAAELSSGKDTPSGHWEIAGVPVLFDWGYFTDKANSFPKELTDRILERAGLDG  141sp|Q2NW04|DEOB_SODGM        EQSTG-RFP-AGLD--KQAEIVGAY-AYASEISSGKDTLSGHWEIAGVPVLFDWGYFPAVENSFPPKLLEALVARAGLPG  140sp|B2VH52|DEOB_ERWT9        EASSG-KFP-PGLD--KNAEIIGAY-AYASELSSGKDTPSGHWEIAGVPVLFDWGYFSDTENSFPQELLDLLVEKANLPG  140sp|A7MGA8|DEOB_CROS8        EGATG-FIP-AGMD--GNAEITGAY-AWAHELSSGKDTPSGHWEIAGVPVLFDWGYFTDEKNSFPQELLDKLVERANLPG  140sp|A4W6A0|DEOB_ENT38        EGSTG-KVA-AGMD--ANAEVVGAY-AWAHELSSGKDTPSGHWEIAGVPVLFDWGYFSDHENSFPQKLLDKLVERGNLPG  140sp|Q327L3|DEOB_SHIDS        EGSTG-FIP-AGMD--GNAEVIGAY-AWAHEMSSGKDTPSGHWEIAGVPVLFEWGYFSDHENSFPQELLDKLVERANLPG  140sp|Q1R260|DEOB_ECOUT (3)    EGSTG-FIP-AGMD--GNAEVIGAY-AWAHEMSSGKDTPSGHWEIAGVPVLFEWGYFSDHENSFPQELLDKLVERANLPG  140sp|B7UR11|DEOB_ECO27        EGSTG-FIP-AGMD--GNAEVIGAY-AWAHEMSSGKDTPSGHWEIAGVPVLFEWGYFSDHENSFPQELLDKLVERANLPG  140sp|Q3YU10|DEOB_SHISS        EGSTG-FIP-AGMD--GNAEVIGAY-AWAHEMSSGKDTPSGHWEIAGVPVLFEWGYFSDHENSFPQELLDKLVERANLPG  140sp|Q0SX28|DEOB_SHIF8 (22)   EGSTG-FIP-AGMD--GNAEVIGAY-AWAHEMSSGKDTPSGHWEIAGVPVLFEWGYFSDHENSFPQELLDKLVERANLPG  140sp|B5Y275|DEOB_KLEP3        EGSTG-KIA-AGMD--GNAEVIGAY-AWAHELSSGKDTPSGHWEIAGVPVLFDWGYFSDHENSFPQELLDKLVKRANLPG  140sp|A9MRA5|DEOB_SALAR        EGSTG-KIA-AGMD--GNADVIGAY-AWAHELSSGKDTPSGHWEIAGVPVLFDWGYFSDHENSFPQELLDKLVKRANLPG  140sp|B5BAJ9|DEOB_SALPK (2)    EGSTG-KIA-AGMD--GNADVIGAY-AWAHELSSGKDTPSGHWEIAGVPVLFDWGYFSDHENSFPQELLDKLVKRANLPG  140sp|C0Q7M5|DEOB_SALPC (2)    EGSTG-KIA-AGMD--GNADVIGAY-AWAHELSSGKDTPSGHWEIAGVPVLFDWGYFSDHENSFPQELLDKLVKRANLPG  140sp|B5R9V1|DEOB_SALG2        EGSTG-KIA-AGMD--GNADVIGAY-AWAHELSSGKDTPSGHWEIAGVPVLFDWGYFSDHENSFPQELLDKLVKRANLPG  140sp|B5FTC7|DEOB_SALDC        EGSTG-KIA-AGMD--GNADVIGAY-AWAHELSSGKDTPSGHWEIAGVPVLFDWGYFSDHENSFPQELLDKLVKRANLPG  140sp|P63924|DEOB_SALTI (8)    EGSTG-KIA-AGMD--GNADVIGAY-AWAHELSSGKDTPSGHWEIAGVPVLFDWGYFSDHENSFPQELLDKLVKRANLPG  140sp|C5BHJ4|DEOB_EDWI9        LESTG-RFP-EGLD--ENAEVIGAY-GYANELSSGKDTPSGHWEIAGVPVLFDWGYFHEHQNSFPQALLDTLVERANLPG  140sp|Q6D990|DEOB_PECAS        EASTG-TFP-AGLD--ENADIIGAY-AHASEISSGKDTPSGHWEIAGVPVLFDWGYFKDEENSFPQELLDKLVKRANLPG  140sp|C6DKL9|DEOB_PECCP        EASTG-TFP-AGLD--ENADIIGAY-AHASEISSGKDTPSGHWEIAGVPVLFDWGYFKDEENSFPQDLLDKLVKRANLPG  140sp|B4EWA2|DEOB_PROMH        EESTG-KFP-VGLD--KDAEIIGAY-GYASEISSGKDTPSGHWEIAGVPVLFDWGYFKDLKNSFPQELLDNIVKRANLPG  141sp|Q7N931|DEOB_PHOLL        EESCG-TFP-AGLD--KDADIIGAY-AYASELSSGKDTPSGHWEIAGVPVLFDWGYFKDEENSFPQALLDKLVERANLPG  140sp|A8G9H8|DEOB_SERP5        EESTG-TFP-QGLD--RNADIIGAY-AHASELSSGKDTPSGHWEIAGVPVLFDWGYFSDEHNSFPQELLDKLVERANLPG  140sp|A1JJ99|DEOB_YERE8        EESTG-KFP-VGLD--KNADIIGAY-GYASELSSGKDTPSGHWEIAGVPVLFDWGYFSDVENSFPQELLDKLVKRANLPG  140sp|A9R047|DEOB_YERPG        EESTG-TFP-VGLD--KNADIIGAY-GYASELSSGKDTPSGHWEIAGVPVLFDWGYFSDVENSFPQELLDKLVKRANLSG  140sp|A4TQJ1|DEOB_YERPP (4)    EESTG-TFP-VGLD--KNADIIGAY-GYASELSSGKDTPSGHWEIAGVPVLFDWGYFSDVENSFPQELLDKLVKRANLPG  140sp|B1JL35|DEOB_YERPY (4)    EESTG-TFP-VGLD--KNADIIGAY-GYASELSSGKDTPSGHWEIAGVPVLFDWGYFSDVENSFPQELLDKLVKRANLPG  140Consensus                   YLGNCHASGTVILDQLGEEHMKTGKPIFYTSADSVFQIACHEETFGLDRLYELCEIAREELEP--YNIGRVIARPFIGDK  221deoB_Bcereus_WP_098782964.1 IIGNKPASGTEILDELGQEQMETGSLIVYTSADSVLQIAAHEEVVPLDELYKICKIARELTLDEKYMVGRVIARPFVGE-  202deoB_Rel606                 YLGNCHSSGTVILDQLGEEHMKTGKPIFYTSADSVFQIACHEGTFGLDKLYELCEIAREELTNGGYNIGRVIARPFIGDK  220sp|Q89A57|DEOB_BUCBP        FLGNCHASGTDILDRFGEIHISTKKPILYTSIDSVCQIACHESIFGLKRLYNLCRSIRKIFDKRKINIARIIARPFTGFK  221sp|Q8K936|DEOB_BUCAP        ILGNCHASGTEIIKILGEEHIKTGKPIFYTSCDSVFQIACHEIKFGLSNLYKICENIREILDQNKYQVARVIARPFIGTN  222sp|B8D866|DEOB_BUCAT        FIGNCHASGTDIISRLGEEHIQTKKPIVYTSSDSVFQIACHEEFFGLSNLYKLCKTVRFILDRYNYKVARVIARPFIGND  220sp|P57607|DEOB_BUCAI        FIGNCHASGTDIISRLGEEHIQTKKPIVYTSSDSVFQIACHEEFFGLSNLYKLCKTVRFILDRYNYKVARVIARPFIGND  220sp|B8D9W4|DEOB_BUCA5        FIGNCHASGTDIISRLGEEHIQTKKPIVYTSSDSVFQIACHEEFFGLSNLYKLCKTVRFILDQYNYKVARVIARPFIGND  220sp|Q483R0|DEOB_COLP3        ILGNCHASGTEILTRLGQEHIETGLPICYTSADSVFQIAAHEEHFGLDNLYKYCETVRELLGD--LNIGRVIARPFVGDS  212sp|Q3ICU9|DEOB_PSET1        ILGNCHASGTTILEQLGEEHVKTGKPICYTSADSVFQIAAHEESFGLEKLYQVCETARSLLDE--MNIGRVIARPFLGSN  212sp|Q5QXT9|DEOB_IDILO        YLGNCHASGTEILKQLGEQHIRSSYPIFYTSADSVFQIAAHEEHFGLERLYQVCEKVRALIEP--YNIGRVIARPFVGDK  211sp|Q17YP1|DEOB_HELAH        YLGNCHASGTEIIKDLGEKHLETLYPIFYTSADSVFQIAVHEEKFGLDKLYALCEEVFEILEP--LKIARVIARSFIGTN  220sp|Q9ZK37|DEOB_HELPJ        YLGNCHASGTEIIKDLGEKHLETLYPIFYTSADSVFQIAAHEERFGLDHLYALCEEAFQILEP--LKIARVIARPFIGTN  220sp|B6JN18|DEOB_HELP2        YLGNCHASGTEIIKDLGEKHLETLYPIFYTSADSVFQIAAHEEKFGLDNLYALCEEAFQILEP--LKIARVIARPFIGTN  220sp|P56195|DEOB_HELPY        YLGNCHASGTEIIKDLGEKHLETLYPIFYTSADSVFQIAAHEERFGLDNLYALCEEAFQILEP--LKIARVIARPFIGTN  220sp|B5Z8H3|DEOB_HELPG        YLGNCHASGTEIIKDLGEKHLETLYPIFYTSADSVFQIAAHEEKFGLDNLYALCEEVFQILEP--LKIARVIARPFIGAN  220sp|B2UUU2|DEOB_HELPS        YLGNCHASGTEIIKDLGEKHLETLYPIFYTSADSVFQIVAHEEKFGLDNLYALCEEAFQILEP--LKIARVIARPFIGAN  220sp|Q1CS87|DEOB_HELPH        YLGNCHASGTEIIKDLGEKHLETLYPIFYTSADSVFQIAVHEEKFGLDNLYALCEEAFQILEP--LKIARVIARPFIGAN  220sp|A4YPV1|DEOB_BRASO        ILGNRHASGTDIISDLGEAHLQTGQPICYTSADSVFQIAAHEEAFGLDRLYRLCEATRPLVDR--FNIGRVIARPFVGRS  218sp|B8IJW7|DEOB_METNO        ILGDCHASGTAIIEELGAEQVRTGKPICYTSVDSVFQIAAHEEAFGLERLSAVCAVARRLCDP--YRIGRVIARPFTGDA  220sp|B0UPC1|DEOB_METS4        ILGDCHASGTAVIEAYGAEHLRSGKPICYTSVDSVFQIAAHEEAFGLERLYALCGIARRLCDP--YRIGRVIARPFAGSA  220sp|B1LYJ9|DEOB_METRJ        ILGDCHAPGVAIIDALGAEHLRTGRPICYTSADSVFQIAAHEEAFGLERLYDLCRVARRLCDR--YRVCRVIARPFVGSP  220sp|B1ZIG8|DEOB_METPB        ILGDRHASGTAIIDALGAEHVRTGRPICYTSADSVFQIAAHEEAFGLEQLYETCRIARAVCDP--YRVGRVIARPFLGSE  220sp|A9W427|DEOB_METEP        ILGDCHASGTAIIEALGAEHVRTGKPICYTSADSVFQIAAHEEAFGLERLYETCRIAREVCDP--YRVGRVIARPFLGSA  220sp|B7KXI3|DEOB_METC4        ILGDCHASGTAIIEALGAEHVRTGKPICYTSADSVFQIAAHEEAFGLERLYETCRIAREVCDP--YRVGRVIARPFLGSA  220sp|C3MBH5|DEOB_SINFN        ILGNCHASGTDIIARHGEEHMRSGKPICYTSSDSVFQIAAHERTFGLERLLELCQVVRRLVDD--YNIGRVIARPFVGDN  220sp|Q92T47|DEOB_RHIME        ILGNCHASGTDIIARHGEEHMRSGKPICYTSSDSVFQIAAHEQTFGLERLLNLCEVVRRLVDD--YNIGRVIARPFVGSD  220sp|A6UET6|DEOB_SINMW        ILGNCHASGTDIIARLGEEHMRTGKPICYTSSDSVFQIAAHEQTFGLERLQDLCAVVRRLVDE--YNIGRVIARPFVGSD  220sp|B9JYP7|DEOB_AGRVS        ILGNCHASGTDILARLGEEHCRTGQPICYTSSDSVFQIAAHEHVFGLERLLRLCEIVRELLTP--YRIGRVIARPFIGNS  220sp|Q8UJ04|DEOB_AGRFC        ILGNCHASGTEIIAALGEEHIRSGKPICYTSSDSVFQIAAHETHFGLDRLIALCETVRKLLDP--LNIGRVIARPFIGET  220sp|B9J6V9|DEOB_AGRRK        ILGNCHASGTDIIARYGEEHIRSGKPICYTSSDSVFQIAAHEQHFGLERLISLCQIVRTLLDP--YNIGRVIARPFIGET  220sp|Q2KDR4|DEOB_RHIEC        ILGNCHASGTEIIARLGEEHIRTGKPICYTSSDSVFQVAAQEAHFGLDRLLTFCRIARGLLDP--YNIGRIIARPFIGQS  220sp|B5ZXI4|DEOB_RHILW        ILGNCHASGTEIIARLGEEHIRTGKPICYTSSDSVFQVAAHEVHFGLDRLLAFCQVARGLLDR--YNIGRVIARPFIGHS  220sp|Q1MMV6|DEOB_RHIL3        ILGNCHASGTEIIARLGEEHIRTGKPICYTSSDSVFQVAAHEVHFGLDRLLAFCGLARGLLDS--YNIGRVIARPFIGQS  220sp|Q98BG5|DEOB_RHILO        ILGNCHAPGTEIIERFGEEHIRTGKPICYTSVDSVLQIAAHEVHFGLDRLYEFCQVVRRLVDP--LRIGRVIARPFVGET  220sp|Q11AV9|DEOB_CHESB        ILANCHASGTEVIARLGEEHIRTGKPICYTSADSVFQIAAHETHFGLDRLYAVCETARRLVDD--YRIGRVIARPFVGES  217sp|A5IGR8|DEOB_LEGPC        VLGEKHASGTIIIDELGEEHIRTGKPIVYTSADSVFQIAAHEEAFGLQRLYDICKIARNLVDK--YQIGRVIARPFAG-K  220sp|Q5X7B2|DEOB_LEGPA        VLGEKHASGTIIIDELGEEHIRTGKPIVYTSADSVFQIAAHEEAFGLQRLYDICKIARNLVDK--YQIGRVIARPFTG-K  220sp|Q5WYR0|DEOB_LEGPL        VLGEKHASGTIIMDELGEEHIRTGKPIVYTSADSVFQIAAHEEAFGLQRLYDICKIARNLVDK--YQIGRVIARPFTG-K  220sp|Q5ZXU2|DEOB_LEGPH        VLGEKHASGTIIIDELGEKHIRTGKPIVYTSADSVFQIAAHEEAFGLQRLYDICKIARNLVDK--YQIGRVIARPFTG-K  220sp|B2KBT7|DEOB_ELUMP        ILGNKAASGTEILEELGEEHIKTGKPICYTSADSVFQIAAHEKHFGLERLYTICEIAFKYLKP--YKIARVIARPFEGER  211sp|Q2SHN3|DEOB_HAHCH        VLGNVHASGTQIIKDLGMEHRATGKPIFYTSADSVVQIACHEETFGLQRLYELCQITRKHIDP--YNIARVIARPFVGAS  219sp|Q1QZC2|DEOB_CHRSD        VLGNCHASGMPILETLGEAHIASGKPIVYTSADSVFQIAAHETHFGLDRLYALCEIARELLMP--YNIGRVIARPFVGET  212sp|A1SYK3|DEOB_PSYIN        YLGNCHASGTTILEDLGEEHLKTGKPIFYTSADSVFQIACHEETFGLERLYKLCELTRELVNK--YNIGRVIARPFSGSA  228sp|A6VM02|DEOB_ACTSZ        YLGNCHSSGTVILDQLGEEHMKTGLPIFYTSADSVFQIAAHEETFGLNNLYELCEIVRTELEG--YNIGRVIARPFIGNK  208sp|C4LAY9|DEOB_TOLAT        YLGNCHASGTEILDRLGEEHMRSGKPIFYTSADSVFQIACHEETYGLDKLYELCKLARQLLEP--YNIGRVIARPFVGSK  218sp|A4SRU2|DEOB_AERS4        YLGNCHASGTQVLDDLGEEHMSTGKPILYTSADSVFQIACHEETYGLEKLYELCHIVRELLEP--YNIGRVIARPFVGSG  216sp|A0KPE2|DEOB_AERHH        YLGNCHASGTQVLDDLGEEHMRTGKPILYTSADSVFQIACHEETYGLEKLYELCHIVRELLEP--YNIGRVIARPFVGSG  216sp|Q7NRT1|DEOB_CHRVO        YLGNCHASGTEILDRLGEEHMKTGKPIFYTSADSVFQIACHEETYGLERLYELCKITRELLEP--YNIGRVIARPFVGEG  217sp|Q086F8|DEOB_SHEFN        FLGNCHASGTQILEELGEEHMKTGKPIFYTSADSVFQIACHEESFGVENLYNLCKIAREELEP--YNIGRVIARPFVGTG  218sp|Q12QG1|DEOB_SHEDO        FLGNCHASGTTILEQLGEEHMRSGMPIFYTSADSVFQVACHEESFGLDNLLRLCEIAREELGP--YNIGRVIARPFIGTG  218sp|A1S476|DEOB_SHEAM        FLGNCHASGTTILEELGEEHMKTGKPIFYTSADSVFQIACHEESFGLENLYRLCEIAREELEP--YNIGRVIARPFVGTG  218sp|Q8EHK2|DEOB_SHEON        FLGNCHASGTTILEELGEEHMRSGKPIFYTSADSVFQIACHEGTFGLENLYRLCEIAREELEP--YNIGRVIARPFDGTG  218sp|A0KU09|DEOB_SHESA        FLGNCHASGTTILEELGEEHMRSGKPIFYTSADSVFQIACHEGTFGLENLYRLCEIAREELEP--YNIGRVIARPFDGTG  218sp|Q0HLE8|DEOB_SHESM        FLGNCHASGTTILEELGEEHMRSGKPIFYTSADSVFQIACHEGTFGLENLYRLCEIAREELEP--YNIGRVIARPFDGTG  218sp|Q0HXQ2|DEOB_SHESR        FLGNCHASGTTILEELGEEHMRSGKPIFYTSADSVFQIACHEGTFGLENLYRLCEIAREELEP--YNIGRVIARPFDGTG  218sp|A1RH89|DEOB_SHESW        FLGNCHASGTTILEELGEEHMRSGKPIFYTSADSVFQIACHEETFGLDNLYRLCEITREELAP--YNIGRVIARPFNGTG  218sp|A4Y9A6|DEOB_SHEPC        FLGNCHASGTTILEELGEEHMRSGKPIFYTSADSVFQIACHEETFGLDNLYRLCEITREELAP--YNIGRVIARPFNGTG  218sp|A6WRB6|DEOB_SHEB8        FLGNCHASGTTILEELGEEHMRSGKPIFYTSADSVFQIACHEETFGLDNLYRLCEITREELEP--YNIGRVIARPFDGTG  218sp|A9KZ78|DEOB_SHEB9 (2)    FLGNCHASGTTILEELGEEHMRSGMPIFYTSADSVFQIACHEETFGLDNLYRLCEITREELEP--YNIGRVIARPFDGTG  218sp|B8E6P6|DEOB_SHEB2        FLGNCHASGTTILEELGEEHMRSGKPIFYTSADSVFQIACHEETFGLDNLYRLCAITREELEP--YNIGRVIARPFDGTG  218sp|B0TQ89|DEOB_SHEHH        FLGNCHSSGTIILEELGEEHMRTGKPIFYTSADSVFQIACHEESFGLEKLYELCIIAREELAD--YNIGRVIARPFVGTG  218sp|A8H726|DEOB_SHEPA        FLGNCHSSGTVILDELGEEHMRTGKPIFYTSADSVFQIACHEESFGLEKLYELCIIAREELAD--YNIGRVIARPFVGTG  218sp|A8FYQ7|DEOB_SHESH        YLGNCHASGTAILEELGNEHMTSGLPIFYTSADSVFQVACHEETFGLENLYTLCQIVREELEP--YNIGRVIARPFVGTG  218sp|A3QGT1|DEOB_SHELP        FLGNCHASGTAILEELGEEHMTSGLPIFYTSADSVFQIACHEETFGLENLYTLCQIAREELEP--YNIGRVIARPFVGTG  218sp|B1KRP6|DEOB_SHEWM        YLGNCHASGTAILEELGEEHMRTGKPIFYTSADSVFQIACHEESFGLENLYNLCIIAREELEP--YNIGRVIARAFVGTG  218sp|Q6LUH2|DEOB_PHOPR        FLGNCHASGTHVLDELGEEHMKTGMPIFYTSADSVFQIACHEETFGLDNLLTLCQIAREELED--YNIGRVIARPFTGPG  219sp|B6ENG5|DEOB_ALISL        YLGNCHASGTQVLDDLGEEHMKTGMPIFYTSADSVFQIACHEETFGLDNLLTLCQIAREELED--YNIGRVIARPFIGAG  219sp|B5FAA0|DEOB_ALIFM        YLGNCHASGTQVLDDLGEEHMKTGMPIFYTSADSVFQIACHEETFGLDNLLTLCQIAREELED--YNIGRVIARPFIGPG  219sp|Q5E7J5|DEOB_ALIF1        YLGNCHASGTQVLDDLGEEHMKTGMPIFYTSADSVFQIACHEETFGLDNLLTLCQIAREELED--YNIGRVIARPFIGPG  219sp|C3LQC0|DEOB_VIBCM (2)    FLGNCHASGTQVLDDLGEEHMRTGKPIFYTSADSVFQIACHEETFGLARLLELCQIAREELAD--YNIGRVIARPFVGPG  219sp|Q9KPL9|DEOB_VIBCH        FLGNCHASGTQVLDDLGEEHMRTGKPIFYTSADSVFQIACHEETFGLDRLLELCQIAREELAD--YNIGRVIARPFVGPG  219sp|A7MUW4|DEOB_VIBCB        FLGNCHASGTQVLDDLGEEHMKTGKPIFYTSADSVFQIACHEETFGLDRLLELCQIAREELED--YNIGRVIARPFVGAG  219sp|Q7MI40|DEOB_VIBVY (2)    FLGNCHASGTQVLDDLGEEHMKTGQPIFYTSADSVFQIACHEETFGLDRLLELCQIAREELAD--YNIGRVIARPFIGPG  219sp|Q87M24|DEOB_VIBPA        FLGNCHASGTQVLDDLGEEHMKTGQPIFYTSADSVFQIACHEETFGLDRLLELCQIAREELED--YNIGRVIARPFIGPG  219sp|Q2NW04|DEOB_SODGM        FLGNCHASGTVILDRLGEEHMRTGKPIFYTSADSVFQLACHEETFGLERLYSLCEIAREILTDGGYNIGRVITRPFVGAK  220sp|B2VH52|DEOB_ERWT9        YLGNCHSSGTVILDQLGAEHMKSGKPIFYTSADSVFQIACHEETFGLERLYALCEIAREALTEGGYNIGRVIARPFVGDK  220sp|A7MGA8|DEOB_CROS8        YLGNCHSSGTVILDELGEEHMKTGKPIFYTSADSVFQIACHEETFGLDRLYELCEIAREELTEGGYNIGRVIARPFVGDK  220sp|A4W6A0|DEOB_ENT38        YLGNCHSSGTVILDQLGEEHMKTGKPIFYTSADSVFQIACHEETFGLDRLYELCEIAREELTEGGYNIGRVIARPFIGNK  220sp|Q327L3|DEOB_SHIDS        YLGNCHSSGTVILDQLGEEHMKTGKPIFYTSADSVFQIACHEETFGLDKLYELCEIAREELINGGYNIGRVIARPFIGDK  220sp|Q1R260|DEOB_ECOUT (3)    YLGNCHSSGTVILDQLGEEHMKTGKPIFYTSADSVFQIACHEETFGLDKLYELCEIAREELTNGGYNIGRVIARPFIGDK  220sp|B7UR11|DEOB_ECO27        YLGNCHSSGTVILDQLGEEHMKTGKPIFYTSADSVFQIACHEETFGLDKLYKLCEIAREELTNGGYNIGRVIARPFIGDK  220sp|Q3YU10|DEOB_SHISS        YLGNCHSSGTVILDQLGEEHMKTGKPIFYTSADSVFQIACHEETFGLDKLYELCEIAREELTNGGYNIGRVIARPFIGDK  220sp|Q0SX28|DEOB_SHIF8 (22)   YLGNCHSSGTVILDQLGEEHMKTGKPIFYTSADSVFQIACHEETFGLDKLYELCEIAREELTNGGYNIGRVIARPFIGDK  220sp|B5Y275|DEOB_KLEP3        YLGNCHSSGTVILDQLGEEHMKTGKPIFYTSADSVFQIACHEETFGLDKLYELCEIAREELTEGGYNIGRVIARPFIGDK  220sp|A9MRA5|DEOB_SALAR        YLGNCHSSGTVILDQLGEEHMKTGKPIFYTSADSVFQIACHEETFGLDKLYELCEIAREELTEGGYNIGRVIARPFIGDK  220sp|B5BAJ9|DEOB_SALPK (2)    YLGNCHSSGTVILDQFGEEHMKTGKPIFYTSADSVFQIACHEETFGLDKLYELCEIAREELTEGGYNIGRVIARPFIGDK  220sp|C0Q7M5|DEOB_SALPC (2)    YLGNCHSSGTVILDQLGEEHMKTGKPIFYTSADSVFQIACHEETFGLDKLYELCEIAREELTEGGYNIGRVIARPFIGDK  220sp|B5R9V1|DEOB_SALG2        YLGNCHSSGTVILDQLGEEHMKTGKPIFYTSADSVFQIACHEETFGLDKLYELCEIAREELTEGGYNIGRVIARPFIGDK  220sp|B5FTC7|DEOB_SALDC        YLGNCHSSGTVILDQLGEEHMKTGKPIFYTSADSVFQIACHEETFGLDKLYELCEIAREELTVGGYNIGRVIARPFIGDK  220sp|P63924|DEOB_SALTI (8)    YLGNCHSSGTVILDQLGEEHMKTGKPIFYTSADSVFQIACHEETFGLDKLYELCEIAREELTEGGYNIGRVIARPFIGDK  220sp|C5BHJ4|DEOB_EDWI9        YLGNCHSSGTVILDQLGEEHMKSGKPIFYTSADSVFQIACHEETFGLERLYELCEIARDELNKGGYNIGRVIARPFVGDK  220sp|Q6D990|DEOB_PECAS        YLGNCHSSGTVILDQLAEEHMKTGKPIFYTSADSVFQIACHEETFGLDKLYELCEIAREELTEGDYNIGRVIARPFIGDK  220sp|C6DKL9|DEOB_PECCP        YLGNCHSSGTVILDQLAEEHMKTGKPIFYTSADSVFQIACHEETFGLDKLYELCEIAREELTEGGYNIGRVIARPFIGDK  220sp|B4EWA2|DEOB_PROMH        YLGNCHASGTVILDELGEEHMKTGKPIFYTSADSVFQIACHEETFGLDKLYELCEIARDELNKGDYNIGRVIARPFIGDK  221sp|Q7N931|DEOB_PHOLL        YLGNCHSSGTVILDKLGEEHMKTGKPIFYTSADSVFQIACHEETFGLDRLYELCEIAREELTDGGYNIGRVIARPFVGDK  220sp|A8G9H8|DEOB_SERP5        YLGNCHSSGTVILDQLGEEHMKTGKPIFYTSADSVFQIACHEETFGLDRLYELCEIAREELTEGGYNIGRVIARPFLGDK  220sp|A1JJ99|DEOB_YERE8        YLGNCHSSGTVILDQLGEEHMKTGKPIFYTSADSVFQIACHEETFGLDRLYELCEIAREELTEGGYNIGRVIARPFIGDK  220sp|A9R047|DEOB_YERPG        YLGNCHSSGTVILDQLGEEHMKTGKPIFYTSADSVFQIACHEETFGLDRLYELCEIAREELTDGGYNIGRVIARPFIGDK  220sp|A4TQJ1|DEOB_YERPP (4)    YLGNCHSSGTVILDQLGEEHMKTGKPIFYTSADSVFQIACHEETFGLDRLYELCEIAREELTDGGYNIGRVIARPFIGDK  220sp|B1JL35|DEOB_YERPY (4)    YLGNCHSSGTVILDQLGEEHMKTGKPIFYTSADSVFQIACHEETFGLDRLYELCEIAREELTDGGYNIGRVIARPFIGDK  220Consensus                   PGNFQRTGNRRDYAVEPPAPTVLDKL-DEKGGEVVSIGKIADIYAHCGITKKVKATGLDALFDATLE----EMKEA--GD  294deoB_Bcereus_WP_098782964.1 PGNFTRTPNRHDYALKPFGRTVMNEL-KDSDYDVIAIGKISDIYDGEGVTESLRTKSNMDGMDKLVD----TLNM----D  273deoB_Rel606                 AGNFQRTGNRHDLAVEPPAPTVLQKLVDEKHGQVVSVGKIADIYANCGITKKVKATGLDALFDATIK----EMKEA--GD  294sp|Q89A57|DEOB_BUCBP        KEHFRRTGNRRDFSMEPHKITVMEKLIGEKKGRVIAIGKISDIYAGKGISCSMYATGLVNLFNTTIQ----EIKNA--KN  295sp|Q8K936|DEOB_BUCAP        KSNFNRTGNRRDISMKPLSITFMEKLIQEKQGKVIAIGKISDIYAGVGISKNIKSTGLYELCHTTIH----QMKSA--KN  296sp|B8D866|DEOB_BUCAT        KLQFQRTGNRRDFSIKPFATTVIKKLIDEKQGQVIAIGKVSDIYGGIGISKNIKSTGLYELCSTTIH----EMKKA--LN  294sp|P57607|DEOB_BUCAI        KLQFQRTGNRRDFSIKPFATTVIKKLIDEKQGQVIAIGKVSDIYGGIGISKNIKSTGLYELCSTTIH----EMKKA--LN  294sp|B8D9W4|DEOB_BUCA5        KLQFQRTGNRRDFSIKPFATTVIKKLIDEKQGQVIAIGKVSDIYGGIGISKNIKSTGLYELCSTTIH----EMKKA--LN  294sp|Q483R0|DEOB_COLP3        ADNFARTGNRRDYSVLPPAPTVLDKI-SQEGTHVISVGKIADIFAHQGIDEKTKATGLNALFDATLD----HINTA--QD  285sp|Q3ICU9|DEOB_PSET1        NQDFARTSNRRDYSVLPPAPTLLDVL-AKDGGEVISIGKISDIYAHQGITQKHKAPGLINLLKKTNE----LMQSA--PD  285sp|Q5QXT9|DEOB_IDILO        ADNFERTANRKDYSVLPPKPTVLDKL-QNKGGKVIAIGKISDIFAGQGVSESVKASGLNGLLTATLN----AMEKA--PE  284sp|Q17YP1|DEOB_HELAH        KDNFKRTSNRKDYAIKPHKKLLFETFIEEKQGEVISIGKIADIYAHVGITQKFKAGSLMELCDVTLE----QVKNA--QN  294sp|Q9ZK37|DEOB_HELPJ        RENFKRTANRKDYAIKPHKKLLFETFIEEKQGEVISIGKIADIYAHVGITQKFKAGSLMELCDVTLD----QVKNA--PN  294sp|B6JN18|DEOB_HELP2        RESFKRTANRKDYAIKPHKKLLFETFIEEKQGEVISIGKIADIYAHVGITQKFKAGSLMELCDVTLE----QVKNA--KN  294sp|P56195|DEOB_HELPY        RESFKRTANRKDYAIKPHKKLLFETFIEEKRGEVISIGKIADIYAHVGITQKFKAGSLMELCDVTLE----QVKNA--KN  294sp|B5Z8H3|DEOB_HELPG        REDFKRTAKRKDYAIKPHKKLLFEKFIEEKQGEVISIGKIADIYAHVGITQKFKAGSLMELCDVTLD----QVKNA--KN  294sp|B2UUU2|DEOB_HELPS        REDFKRTAHRKDYAIKPHKKLLFEKFIEEKQGEVISIGKIADIYAHVGITQKFKAGSLMELCDVTLE----QIKNA--KN  294sp|Q1CS87|DEOB_HELPH        REDFKRTANRKDYAIKPHKKLLFETFIEEKQGEVISIGKIADIYAHVGITQKFKAGSLMELCDVTLE----QIKNA--KN  294sp|A4YPV1|DEOB_BRASO        ASGFRRTANRRDYAVPPPEPTILDLA-TAERRHVVTIGKIADIFAHRGTGHNLKGDSNDTLFTRMLE----GLDAL--DD  291sp|B8IJW7|DEOB_METNO        RRGFVRTANRRDFATPPPSDTLLDRL-VAAGRPVVSVGKIGDIFAHRSTGSEVKPAGNEACLDAALA----AFAESGLGP  295sp|B0UPC1|DEOB_METS4        GTGFVRTANRRDFATPPPGDTLLDRL-AAAGRPLVSVGKIGDIFAHRHTGTEVKPAGNDACLDAALA----AFADL--GP  293sp|B1LYJ9|DEOB_METRJ        EAGFRRTGNRRDLAVAPPGRTLLDRA-EAAGRAVVSVGKIGDIFAHRATGREIKPGPNAACLTAGLD----ALATL--PQ  293sp|B1ZIG8|DEOB_METPB        VDGFRRTSRRKDFSVAPPDGTLLDGL-EAAGRAIVSVGKIGDIFAHRATGREIKPAGNAACLDAALD----AFAEL--PE  293sp|A9W427|DEOB_METEP        AEGFRRTSHRKDFSVAPPAGTLLDGL-ETAGRAVVSVGKIGDIFAHRATGREIKPAGNAACLDAALD----AFAGL--PE  293sp|B7KXI3|DEOB_METC4        AEGFRRTSHRKDFSVAPPAGTLLDGL-EAAGRAVVSVGKIGDIFAHRATGREIKPAGNAACLDAALD----AFAGL--PE  293sp|C3MBH5|DEOB_SINFN        PGNFTRTGHRRDFSVLPPEPTILDRL-EAAGRTVHAIGKIGDIFAHRGVTRLTKANGNMELFDASLT----VVEEA--EE  293sp|Q92T47|DEOB_RHIME        PGSFTRTGNRRDYSVLPPEPTVLDRL-QEAGRTVHAIGKIGDIFAHQGVTRLTKANGNMALFDASLE----AIEEA--ED  293sp|A6UET6|DEOB_SINMW        PGSFTRTGNRRDYSVLPPAPTVLDRL-KEAGRTVHAIGKIADIFAHQGVTRLTKANGNMALFDASLA----AIDEA--ED  293sp|B9JYP7|DEOB_AGRVS        ASNFQRTGNRRDYSVPPPEPTLLDRL-SEAGRTVHAIGKIGDIFAHQGTGRDIKANGNAALMEATLA----VMDEA--AD  293sp|Q8UJ04|DEOB_AGRFC        VATFERTGNRRDFSVPPPEPTLLDRL-VEAGRKVHAIGKIGDIYAHQGVTRVIKANGNAALMDATLH----AIDEA--EN  293sp|B9J6V9|DEOB_AGRRK        PANFERTGNRRDFSVLPPEPTLLDRL-VAAERKVHAVGKIGDIFAHQGISRIIKANGNMKLMDATLK----TMDEA--AD  293sp|Q2KDR4|DEOB_RHIEC        ASTFQRTGNRRDFSVLPPEPTLLDRL-LQHGRHVHAVGKIGDIFAHQGISRVIKATGNEALMDASLS----AIDAA--ED  293sp|B5ZXI4|DEOB_RHILW        SSTFQRTGNRRDFSVLPPEPTLLDRL-IEHGRHVHAVGKIGDIFAHQGISRLIKANGNEALMDATLA----TIDEA--ED  293sp|Q1MMV6|DEOB_RHIL3        ASTFQRTGNRRDFSVLPPEPTLLDRL-IEQGRHVHAVGKIGDIFAHQGISRVIKANGNEALMDASLS----AIDAA--ED  293sp|Q98BG5|DEOB_RHILO        AATFQRTYNRHDYAVPPPEPTLLDRL-TARGSRVIAVGKIGDIFAHRGISEVRKAAGNMAMFDKALG----AMDDA--GD  293sp|Q11AV9|DEOB_CHESB        AETFERTANRRDYAVPPPEPTLLDRV-EAAGRRVIGIGKIGDIFAHQGVTEVRKAAGNMALFDAALG----AMDDA--RE  290sp|A5IGR8|DEOB_LEGPC        PGSFKRTGNRKDYATPPPEKTLLDFL-KEDGREVIAIGKIADIYAHQGVTQEIKADGNMALFDATLS----AMKTA--PQ  293sp|Q5X7B2|DEOB_LEGPA        PGSFKRTGNRKDYATPPPEKTLLDFL-KEDGREVIAIGKIADIYAHQGVTQEIKADGNMALFDATLS----AMKTA--PQ  293sp|Q5WYR0|DEOB_LEGPL        PGSFKRTGNRKDYATPPPEKTLLDFL-KEDGREVIAIGKIADIYAHQGVTQEIKADGNMALFDATLS----AMKTA--PQ  293sp|Q5ZXU2|DEOB_LEGPH        PGSFKRTGNRKDYATPPPEKTLLDFL-KEDGREVIAIGKIADIYAHQGVTQEIKADGNMALFDATLS----AMKTA--PQ  293sp|B2KBT7|DEOB_ELUMP        KGEFKRTKNRHDYAVKPPAPTVLDFL-KENGGNVISIGKINDIYAKQGITKAVKASGLEELWNTTIE----ETKNA--SG  284sp|Q2SHN3|DEOB_HAHCH        PEGFRRTGARRDFATPPHKPTLLDKL-NNHGKQVIAIGKISDIYAGKGVSRSVKADGLGALVEATLTVMN-ETDEAAAKQ  297sp|Q1QZC2|DEOB_CHRSD        PETFERTGNRRDYAIEPPTPTVLQKL-HDDGGKVLGVGKIGDIYAHCGVSNVIKAHGHDALFDATLE----ALDEA--GD  285sp|A1SYK3|DEOB_PSYIN        SSNFMRTGNRHDYSVKPPSPTLLESM-KESGGQVVSIGKISDIFAEQGITKATKANGLEALFDASLN----ELKQA--GD  301sp|A6VM02|DEOB_ACTSZ        AGAFKRTGNRRDYSVEPPAKTVLQKFIEEKEGMVVSVGKIADIYAHTGISKKVKATGLEELFDKTLE----EVKSA--GD  282sp|C4LAY9|DEOB_TOLAT        AGEFKRTGNRHDYAVEPPMPTLLDRM-KAAGGDVISIGKIADIYACCGITQQHKATGLDELWDMTLA----QVKTA--AD  291sp|A4SRU2|DEOB_AERS4        KGNFKRTGNRHDYSVLPPAPTVLDYM-KEAGGQVVSIGKIADIYANKGITKQVKGTGLTELWDRTLE----EVKAA--GD  289sp|A0KPE2|DEOB_AERHH        KGNFKRTGNRHDYSVLPPAPTVLDYM-KEAGGQVVSIGKIADIYAQQGITKQVKGTGLTELWDRTLE----EVKAA--GD  289sp|Q7NRT1|DEOB_CHRVO        KGKFARTGNRKDLAVEPPAATVLKKL-ADAGGDVVSIGKIADIYAHVGITHKHKATGFDQLWDATLT----AMDQH--RD  290sp|Q086F8|DEOB_SHEFN        PADFARTGNRHDYAVLPPAPTVLDKL-KDAGGEVVSIGKISDIYAHSGITQQFKATGLEELFDETLA----QIKRA--GD  291sp|Q12QG1|DEOB_SHEDO        PSDFSRTGNRRDYAVEPPSKTVLDKL-KDAGGEVVSVGKIADIYAHCGITKKVKASGLEALFDATLE----QIKQA--GD  291sp|A1S476|DEOB_SHEAM        PSDFARTGNRRDYAVEPPSKTVLDKL-KQAGGEVVSVGKISDIYAHCGITKKVKASGLEDLFDATLE----QIKQA--GD  291sp|Q8EHK2|DEOB_SHEON        PSDFARTGNRKDYSLEPPAKTVLDKL-KAAGGEVVSVGKIADIYAYCGITKKVKANGLEALFDATLA----EVKSA--GE  291sp|A0KU09|DEOB_SHESA        PSDFARTGNRKDYSLEPPAKTVLDKL-KAAGGEVVSVGKIADIYAYCGITKKVKANGLEALFDATLA----EVKSA--GE  291sp|Q0HLE8|DEOB_SHESM        PSDFARTGNRKDYSLEPPAKTVLDKL-KAAGGEVVSVGKIADIYAYCGITKKVKANGLEALFDATLD----EVKSA--GE  291sp|Q0HXQ2|DEOB_SHESR        PNDFARTGNRKDYSLEPPAKTVLDKL-KAAGGEVVSVGKIADIYAYCGITKKVKANGLEALFDATLA----EVKSA--GE  291sp|A1RH89|DEOB_SHESW        PSDFARTGNRKDYSLEPPAKTVLDKL-KAAGGEVVSVGKIADIYAYCGITKKVKANGLEDLFDATLA----EVKSA--GD  291sp|A4Y9A6|DEOB_SHEPC        QSDFARTGNRKDYSLEPPAKTVLDKL-KAAGGEVVSVGKIADIYAYCGITKKVKANGLEDLFDATLA----EVKSA--GD  291sp|A6WRB6|DEOB_SHEB8        PSDFARTGNRKDYSLEPPAKTVLDKL-KEAGGEVVSVGKIADIYAYCGITKKVKANGLEALFDATLA----EVKSA--GD  291sp|A9KZ78|DEOB_SHEB9 (2)    PSDFARTGNRKDYSLAPPAKTVLDKL-NEAGGEVVSVGKIADIYAYCGITKKVKANGLEALFDATLA----EVKSA--GD  291sp|B8E6P6|DEOB_SHEB2        SSDFARTGNRKDYSLAPPAKTVLDKL-NEAGGEVVSVGKIADIYAYCGITKKVKANGLEALFDATLA----EVKSA--GD  291sp|B0TQ89|DEOB_SHEHH        PSDFARTGNRRDYAVEPPAPTVLDKL-KAAGGEVVSVGKIADIYAHCGITKKVKATGLEELFDATLE----QVKQA--GD  291sp|A8H726|DEOB_SHEPA        PGSFERTGNRRDYAVEPPAPTVLDKL-KAAGGEVVSVGKIADIYAHCGITKKVKATGLEALFDATLE----QVKLA--GD  291sp|A8FYQ7|DEOB_SHESH        PSDFARTGNRRDYAVEPPAKTVLDKL-KDSGGEVVSVGKIADIYAHCGITKKIKASGLEALFDATLE----ELKVA--GD  291sp|A3QGT1|DEOB_SHELP        PSDFARTGNRKDYAVEPPSKTVLDKL-KEAGGEVVSVGKIADIYAHCGITKKVKATGLEALFDATLE----QVKQA--GD  291sp|B1KRP6|DEOB_SHEWM        PSDFARTGNRRDYAVEPPSKTVLDKM-KAAGGEVISVGKIADIYANCGITQKVKATGLEALFDATLE----QVKAA--GD  291sp|Q6LUH2|DEOB_PHOPR        KGQFERTGNRRDLSLEPPATTVLQKLVDEKGGDVISIGKISDIYAGCGITKKVKANGIPALFEATLE----QIKQA--GD  293sp|B6ENG5|DEOB_ALISL        KGQFERTGNRRDLSLEPPAITVLQKLVEEKNGHVHSIGKISDIYAGCGITQKTKATGIPALFDATKE----AITAA--SD  293sp|B5FAA0|DEOB_ALIFM        KGQFERTGNRRDLSVEPPAATILQKLVDEKGGQVHSIGKISDIYAGCGITKKTKATGIPALFDATKE----AIEQA--GD  293sp|Q5E7J5|DEOB_ALIF1        KGQFERTGNRRDLSVEPPAATILQKLVDEKGGQVHSIGKISDIYAGCGITKKTKATGIPALFDATKEAIEQAIEQA--GD  297sp|C3LQC0|DEOB_VIBCM (2)    KGQFARTGNRRDLSVEPPSATVLQKLVEEKQGRVVSIGKIADIYAYCGITDKVKATGIPDLFEATLE----QIKQA--GD  293sp|Q9KPL9|DEOB_VIBCH        KGQFARTGNRRDLSVEPPSATVLQKLVEEKQGRVVSIGKIADIYAYCGITDKVKATGIPDLFEATLE----QIKQA--GD  293sp|A7MUW4|DEOB_VIBCB        KGQFERTGNRRDLSVEPPSATVLQKLVEEKQGDVVSIGKIADIYANCGITKKVKATGIPALFEATLE----QIKEA--GD  293sp|Q7MI40|DEOB_VIBVY (2)    KGQFERTGNRRDLSVEPPSATVLQKLAEEKQGQVVSIGKIADIYANCGITKKVKATGIPALFEATLE----QIKQA--GD  293sp|Q87M24|DEOB_VIBPA        KGQFERTGNRRDLSVEPPSATVLQKLVEEKQGNVVSIGKIADIYANCGITKKVKATGIPALFEATLE----QIKEA--GD  293sp|Q2NW04|DEOB_SODGM        AGQFERTGNRHDLAVPPPSATMLQKLVEEKGGTVVSVGKIADIYAQVGISKKVKATGLDALFDATVR----EMDAA--GE  294sp|B2VH52|DEOB_ERWT9        PGHFERTGNRHDLAVEPPAPTVLKKLVDEQGGEVISVGKIADIYAHVGITKKVKATGLDALFDATVG----EMKSA--PD  294sp|A7MGA8|DEOB_CROS8        AGNFQRTGNRHDLAVEPPSPVVLKKLVDEKGGHVVSVGKIADIYANMGITKKVKATGLDALFDATIK----EMKEA--GD  294sp|A4W6A0|DEOB_ENT38        PGEFQRTGNRHDLAVEPPAATVLQKLVDEKDGQVVSVGKIADIYANCGITKKVKATGLDALFDATVK----EMKEA--GD  294sp|Q327L3|DEOB_SHIDS        AGNFQRTGNRHDLAVEPPAPTVLQKLVDEKHGQVVSVGKIADIYANCGITKKVKATGLDALFDATIK----EMKEA--GD  294sp|Q1R260|DEOB_ECOUT (3)    AGNFQRTGNRHDLAVEPPAPTVLQKLVDEKHGQVVSVGKIADIYANCGITKKVKATGLDALFDATIK----EMKEA--GD  294sp|B7UR11|DEOB_ECO27        AGNFQRTGNRHDLAVEPPAPTVLQKLVDEKHGQVVSVGKIADIYANCGITKKVKATGLDALFDATIK----EMKEA--GD  294sp|Q3YU10|DEOB_SHISS        AGNFQRTGNRHDLAVEPPAPTVLQKLVDEKHGQVVSVGKIADIYANCGITKKVKATGLDALFDATIK----EMKEA--GD  294sp|Q0SX28|DEOB_SHIF8 (22)   AGNFQRTGNRHDLAVEPPAPTVLQKLVDEKHGQVVSVGKIADIYANCGITKKVKATGLDALFDATIK----EMKEA--GD  294sp|B5Y275|DEOB_KLEP3        AGNFQRTGNRHDLAVEPPAPTVLQKLVDEKNGHVVSVGKIADIYANCGITKKVKATGLDALFDATIK----EMKEA--GD  294sp|A9MRA5|DEOB_SALAR        AGNFQRTGNRHDLAVEPPAPTVLQKLVDEKQGHVVSVGKIADIYANCGITKKVKATGLDALFDATIK----EMKDA--GD  294sp|B5BAJ9|DEOB_SALPK (2)    AGNFQRTGNRHDLAVEPPAPTVLQKLVDEKQGHVVSVGKIADIYANCGITKKVKATGLDALFDATLK----EMKEA--GD  294sp|C0Q7M5|DEOB_SALPC (2)    AGNFQRTGNRHDLAVEPPAPTVLQKLVDEKQGHVVSVGKIADIYANCGITKKVKATGLDALFDATLK----EMKEA--GD  294sp|B5R9V1|DEOB_SALG2        AGNFQRTGNRHDLAVEPPAPTVLQKLVDEKQGHVVSVGKIADIYANCGITKKVKATGLDALFDATLK----EMKEA--GD  294sp|B5FTC7|DEOB_SALDC        AGNFQRTGNRHDLAVEPPAPTVLQKLVDEKQGHVVSVGKIADIYANCGITKKVKATGLDALFDATLK----EMKEA--GD  294sp|P63924|DEOB_SALTI (8)    AGNFQRTGNRHDLAVEPPAPTVLQKLVDEKQGHVVSVGKIADIYANCGITKKVKATGLDALFDATLK----EMKEA--GD  294sp|C5BHJ4|DEOB_EDWI9        AGHFQRTGNRHDLAVEPPAPTMLKKLVDEKQGDVVSIGKIADIYANVGITKKVKATGIDALFDATLQ----EMRQA--GN  294sp|Q6D990|DEOB_PECAS        PGNFERTGNRHDLAVEPPAPTILKKLVDEKGGEVVSVGKIADIYAQVGITKKVKATGIDALFDATLK----EMDSA--GD  294sp|C6DKL9|DEOB_PECCP        PGHFERTGNRHDLAVEPPAPTILKKMVDEKGGEVVSVGKIADIYAQVGITKKVKATGIDALFDATLK----EMDSA--GD  294sp|B4EWA2|DEOB_PROMH        PGNFSRTGNRHDLAVEPPAPTMLKKLVDEKQGHVVSIGKIADIYANVGITKKVKATGIDALFDATIE----EMKLA--GD  295sp|Q7N931|DEOB_PHOLL        AGNFQRTGNRHDLAVEPPAPTILKKLVDEKNGEVVSIGKIADIYANVGITQKVKATGIDALFDATLV----EMEKA--GD  294sp|A8G9H8|DEOB_SERP5        PGNFQRTGNRHDLAVEPPAPTVLKKLVDEKGGEVVSIGKIADIYANVGITKKVKATGIDALFDATLI----EMEKA--GD  294sp|A1JJ99|DEOB_YERE8        PGNFQRTGNRHDLAVEPPAPTMLKKLVDEKGGEVVSIGKIADIYAHVGITQKVKATGLDALFDATIE----EMKKA--GD  294sp|A9R047|DEOB_YERPG        PGHFQRTGNRHDLAVEPPAPTMLKKLVDEKGGEVVSIGKIADIYAQVGITQKVKATGLDALFDATIE----EMKKA--GD  294sp|A4TQJ1|DEOB_YERPP (4)    PGHFQRTGNRHDLAVEPPAPTMLKKLVDEKGGEVVSIGKIADIYAQVGITQKVKATGLDALFDATIE----EMKKA--GD  294sp|B1JL35|DEOB_YERPY (4)    PGHFQRTGNRHDLAVEPPAPTMLKKLVDEKGGEVVSIGKIADIYAQVGITQKVKATGLDALFDATIE----EMKKA--GD  294Consensus                   N-TIVFTNFVDFDSSYGHRRDVAGYAAALEYFDXRLPELLALLKEDDLLILTADHGCDPTWPGTDHTREHIPVLAYGPGV  373deoB_Bcereus_WP_098782964.1 FTGLSFLNLVDFDALFGHRRDPQGYGEALQEYDARLPEVFAKLKEDDLLLITADHGNDPIHPGTDHTREYVPLLAYSPSM  353deoB_Rel606                 N-TIVFTNFVDFDSSWGHRRDVAGYAAGLELFDRRLPELMSLLRDDDILILTADHGCDPTWTGTDHTREHIPVLVYGPKV  373sp|Q89A57|DEOB_BUCBP        N-TIVFVNFVDFDSLWGHRRDVSGYAKDLEWFDYNLPKLLKLVHNEDLLIITADHGCDPTWIGTDHTRENVPILIYQRSM  374sp|Q8K936|DEOB_BUCAP        N-TVIFTNFVDFDSVWGHRRDVSGYAKGLEFFDSKLSEMIDLVKEGDLFILTADHGCDPTWKGTDHTRENVPILIYSPGE  375sp|B8D866|DEOB_BUCAT        N-TIVFTNLVDFDSNWGHRRDVSGYAKGLELFDSRLSEIISLVQKNDLLILTADHGCDPTWIGTDHTRENVPVLIYSPGI  373sp|P57607|DEOB_BUCAI        N-TIVFTNLVDFDSNWGHRRDVSGYAKGLELFDSRLSEIISLVQKNDLLILTADHGCDPTWIGTDHTRENVPVLIYSPGI  373sp|B8D9W4|DEOB_BUCA5        N-TIVFTNLVDFDSNWGHRRDVSGYAKGLELFDSRLSEIISLVQKNDLLILTADHGCDPTWIGTDHTRENVPVLIYSPGI  373sp|Q483R0|DEOB_COLP3        N-SLIFTNLVNFDQDFGHRRDAIGYAKELEALDVRIPELFHAMSAEDVLFLTADHGCDPTWPGTEHTREYVPIIAYHHQI  364sp|Q3ICU9|DEOB_PSET1        H-SLIFTNLVDFDEMFGHRRNPVGYAKALKEFDDYLPTILNALKADDLLIITADHGCDPTFPGSEHTREYVPVIAYQPGM  364sp|Q5QXT9|DEOB_IDILO        N-TLIFTNLVDFDTLYGHRRDIDGYARELETFDQWLPVIESAMTPDDVLVLTADHGCDPTWQGTDHTRELIPLLLSGQNV  363sp|Q17YP1|DEOB_HELAH        N-SLIFTNFVHFDSDYGHRRDVIGYANALEYFDAYLKEILESLRENDLLILCADHGCDPSFKGTDHTREYIPVLMYHKDL  373sp|Q9ZK37|DEOB_HELPJ        N-SLIFTNFVHFDSDYGHRRDVSGYANALEYFDARLKEVLDNLRENDLLILCADHGCDPSFKGTDHTREYIPVLFYHKDL  373sp|B6JN18|DEOB_HELP2        N-SLIFTNFVHFDSDYGHRCDISGYANALEYFDTRLKEVLENLRENDLLILCADHGCDPSFKGTDHTREYIPVLFYHKDL  373sp|P56195|DEOB_HELPY        N-SLIFTNFVHFDSDYGHRRDISGYANALEYFDARLKEVLENLRENDLLILCADHGCDPSFKGTDHTREYIPVLFYHKDL  373sp|B5Z8H3|DEOB_HELPG        N-SLIFTNFVHFDSDYGHRRDISGYANALEYFDTRLKEVLENLKENDLLILCADHGCDPSFKGTDHTREYIPVLFYHKDL  373sp|B2UUU2|DEOB_HELPS        N-SLIFTNFVHFDSDYGHRRDISGYANALEYFDARLKEVLENLRENDLLILCADHGCDPSFKGTDHTREYIPVLFYHKDL  373sp|Q1CS87|DEOB_HELPH        N-SLIFTNFVHFDSDYGHRRDISGYANALEYFDARLKEILDNLKENDLLILCADHGCDPSFKGTDHTREYIPVLFYHKDL  373sp|A4YPV1|DEOB_BRASO        G-GLLFANFIDFDTLYGHRRDVAGYASALEAFDARLPELLDALRDDDLLVITADHGCDPSWSGSDHTRERVPVLIVN-GR  369sp|B8IJW7|DEOB_METNO        G-GLVFVNLVDFDTEYGHRRDVPGYAAALERFDARIPEIRAGLAPGDLCVITADHGNDPTWSGTDHTREQVPVLSFGPGL  374sp|B0UPC1|DEOB_METS4        G-GLVFANLVDFDTEYGHRRDVPGYAAALERFDARLPEIRAALRPGDLCLVTADHGNDPTWTGTDHTREQVPVLAFGPGQ  372sp|B1LYJ9|DEOB_METRJ        G-GLIFVNLVDFDTEHGHRRDVPGYAAELEAFDARIPEILAALAPGDLAVITADHGNDPTWTGTDHTREQVPVLAFGPGV  372sp|B1ZIG8|DEOB_METPB        G-GFVFLNLVDFDTEHGHRRDVPGYAAELEAFDARIPEIHAVLRPGDLCVITADHGNDPTWTGTEHTREQVPVLAFGPGL  372sp|A9W427|DEOB_METEP        G-GFVFLNLVDFDTEHGHRRDVPGYAAELEAFDRRLPEIQAVLKPGDLCVITADHGNDPTWTGTEHTREQVPVLAFGPGL  372sp|B7KXI3|DEOB_METC4        G-GFVFLNLVDFDTEHGHRRDVPGYAAELEAFDARLPEIQAVLKPGDLCVITADHGNDPTWTGTEHTREQVPVLAFGPGL  372sp|C3MBH5|DEOB_SINFN        G-DLVFTNFVDFDMLYGHRRDVPGYAAALEAFDARLPDLDRRLRPGDMVILTADHGCDPTWRGTDHTRERVPVLMFGPSL  372sp|Q92T47|DEOB_RHIME        G-ALVFTNFVDFDMLYGHRRDVSGYAAALEAFDARLPDLDRRLKPGDMVILTADHGCDPTWRGTDHTRERVPVLMFGPTL  372sp|A6UET6|DEOB_SINMW        G-ALIFTNFVDFDMLYGHRRDVAGYAAALEAFDARLPDLDRRLKPGDMVILTADHGCDPTWRGTDHTRERVPVLMFGPTL  372sp|B9JYP7|DEOB_AGRVS        G-DLVFTNFVDFDMLYGHRRDVPGYAAALEAFDLWLPDVYRKLIPGDMVILTADHGCDPTWRGTDHTRERVPIMAFGPGI  372sp|Q8UJ04|DEOB_AGRFC        G-DLVFTNFVDFDMLYGHRRDVAGYAAALEAFDARIPEIHRKMAPGDIALLTADHGCDPTWRGTDHTRERVPIMAFGPGI  372sp|B9J6V9|DEOB_AGRRK        G-DLVFTNFVDFDMVYGHRRDVAGYAAALEAFDARLPEVHRKLKPGDLLILTADHGCDPTWRGTDHTRERVPIIAYELGI  372sp|Q2KDR4|DEOB_RHIEC        G-DLVFTNFVDFDMNYGHRRDVPGYAAALEAFDARLPEVHKKLKPGDLVVLTADHGCDPTWRGTDHTRERVPIIAFGPGI  372sp|B5ZXI4|DEOB_RHILW        G-DLVFTNFVDFDMVYGHRRDVPGYAAALEAFDARLAEVHKKLKPGDLVVLTADHGCDPTWRGTDHTRERVPVIAYGPGI  372sp|Q1MMV6|DEOB_RHIL3        G-DLVFTNFVDFDMIYGHRRDVPGYAAALEAFDARLPDVHKKLKPGDLVVLTADHGCDPTWRGTDHTRERVPVIAYGPGI  372sp|Q98BG5|DEOB_RHILO        G-DLVFANFVDFDTEFGHRRDVAGYAAALEAFDRRLPEAFARLRQGDLLILTADHGNDPTWRGTDHTRERIPVIGMGPGL  372sp|Q11AV9|DEOB_CHESB        G-DLVFANFVDFDSLYGHRRDVAGYAAALEAFDRRLPEALGKLRAGDLLILTADHGCDPTWRGTDHTRECVPILGAGPGL  369sp|A5IGR8|DEOB_LEGPC        G-SLVFTNFVDFDSSYGHRRDVAGYAHALEQFDARLPELEVLLQPNDMVFIAADHGCDPTFPGSDHTREHIPVLMFGPQV  372sp|Q5X7B2|DEOB_LEGPA        G-SLVFTNFVDFDSSYGHRRDVAGYAHALEQFDVRLPELEALLQPDDMVFIAADHGCDPTFPGSDHTREHIPVLMFGPQV  372sp|Q5WYR0|DEOB_LEGPL        G-SLVFTNFVDFDSSYGHRRDIAGYAHALEQFDARLPELEVLLQPNDMVFIAADHGCDPTFPGSDHTREHIPVLVFGPQV  372sp|Q5ZXU2|DEOB_LEGPH        G-SLVFTNFVDFDSSYGHRRDIAGYAHALEQFDARLPELDALLQPDDMVFIAADHGCDPTFPGSDHTREHIPVLVFGPQV  372sp|B2KBT7|DEOB_ELUMP        N-SIIFTNFVDFDMVWGHRRDFKGYAGGLEYFDSRLPELANILQEGDLVFITADHGCDPSYKGTDHTRENVPAIMFGKNV  363sp|Q2SHN3|DEOB_HAHCH        D-TLIFTNLVDFDMLYGHRRDVTGYAKALEDFDAMLPDLLGAMMDDDLLILTADHGCDPTWPGSDHTREHIPILMYGKRA  376sp|Q1QZC2|DEOB_CHRSD        R-TLVMTNFVDFDTLYGHRRDPDGYAEALEAFDRRLPEVLAKLRPDDLLILTADHGNDPTWTGTDHTREQVPVLASGAGL  364sp|A1SYK3|DEOB_PSYIN        Q-TIIFTNFVNFDADFGHRRNLPGYAAALEYFDKRLPEMLALMTEDDLLVLTADHGCDPTWKGTDHTREHIPAIFYGHAV  380sp|A6VM02|DEOB_ACTSZ        N-TIVFTNFVNFDADFGHRRDVTGYAKGLEYFDRRLPELLRLMKDDDLLIITADHGCDPTWQGSDHTREHIPVLMYGAQV  361sp|C4LAY9|DEOB_TOLAT        Q-SIIFTNFVDFDSSYGHRRDVAGYAAALEYFDSRLPELLALLLPGDRVVLTADHGCDPIWTGTDHTREHVPVIFYGDTV  370sp|A4SRU2|DEOB_AERS4        N-TIVFTNFVDFDSSYGHRRDVKGYADALEYFDSRLPELFEILQDGDVVVLTADHGCDPTWGGTDHTREYIPVLFFGKPV  368sp|A0KPE2|DEOB_AERHH        Q-TIVFTNFVDFDSSYGHRRDVKGYADALEYFDSRLPELFELLEDGDVVVLTADHGCDPTWGGTDHTREYIPVLFYGKPV  368sp|Q7NRT1|DEOB_CHRVO        KPAIIMANFVDFDSSYGHRRNTAGYAAALEEFDARLPEVMAALGDDDILILSADHGCDPTWPGTDHTREHIPVLCYGKQV  370sp|Q086F8|DEOB_SHEFN        N-TIVFTNFVDFDSHYGHRRDTAGYAKALEYFDSRLPELLAILQPEDLVIFTADHGCDPTWVGTEHTRERVPVLAYGAGL  370sp|Q12QG1|DEOB_SHEDO        R-TIVFTNFVDFDSHYGHRRDVAGYAKALEYFDERLPELLALLEQDDLLLLTADHGCDPTWPGSDHTRERVPVLALGAGL  370sp|A1S476|DEOB_SHEAM        N-TIVFTNFVDFDSHYGHRRDVSGYAKALEYFDARLPELMALLEEGDLLLLTADHGCDPTWQGTDHTREFVPVLAYGAGL  370sp|Q8EHK2|DEOB_SHEON        N-TIVFTNFVDFDSHYGHRRDVAGYAKGLEYFDARLPEMLALLDEDDLLILTADHGCDPTWQGTDHTREYVPVLAYGAGL  370sp|A0KU09|DEOB_SHESA        N-TIVFTNFVDFDSHYGHRRDVAGYAKGLEYFDSRLPEMLALLDEDDLLILTADHGCDPTWQGTDHTREYVPVLAYGAGL  370sp|Q0HLE8|DEOB_SHESM        N-TIVFTNFVDFDSHYGHRRDVAGYAKGLEYFDSRLPEMLALLDEDDLLILTADHGCDPTWQGTDHTREYVPVLAYGAGL  370sp|Q0HXQ2|DEOB_SHESR        N-TIVFTNFVDFDSHYGHRRDVAGYAKGLEYFDSRLPEMLSLLDEDDLLILTADHGCDPTWQGTDHTREYVPVLAYGAGL  370sp|A1RH89|DEOB_SHESW        N-TIVFTNFVDFDSHYGHRRDVAGYAKGLEYFDARLPEMLALLGEDDLLILTADHGCDPTWQGTDHTREYVPVLAYGAGL  370sp|A4Y9A6|DEOB_SHEPC        N-TIVFTNFVDFDSHYGHRRDVAGYAKGLEYFDARLPEMLALLGEDDLLILTADHGCDPTWQGTDHTREYVPVLAYGAGL  370sp|A6WRB6|DEOB_SHEB8        N-TIVFTNFVDFDSHYGHRRDVAGYAKGLEYFDARLPEMLALLGEDDLLILTADHGCDPTWQGTDHTREYVPVLAFGAGL  370sp|A9KZ78|DEOB_SHEB9 (2)    N-TIVFTNFVDFDSHYGHRRDVAGYAKGLEYFDARLPEMLALLGEDDLLILTADHGCDPTWQGTDHTREYVPVLAFGAGL  370sp|B8E6P6|DEOB_SHEB2        N-TIVFTNFVDFDSHYGHRRDVAGYAKGLEYFDARLPEMLALLGEDDLLILTADHGCDPTWQGTDHTREYVPVLAFGAGL  370sp|B0TQ89|DEOB_SHEHH        N-TIVFTNFVDFDSHFGHRRDVAGYARSLEYFDSRLPEILALLDEDDFLLLTADHGCDPTWPGSDHTRERVPVLAYGAGL  370sp|A8H726|DEOB_SHEPA        N-TIVFTNFVDFDSHYGHRRDVAGYARSLEYFDSRLPEILALLDEDDFLLLTADHGCDPTWPGSDHTRERVPVLAYGAGL  370sp|A8FYQ7|DEOB_SHESH        N-TIVFTNFVDFDSHFGHRRDVAGYARSLEYFDSRLPELMALMGEDDLLLLTADHGCDPTWPGTDHTRERVPVLAYGAGL  370sp|A3QGT1|DEOB_SHELP        R-TIVFTNFVDFDSHYGHRRDIAGYARALEYFDSRLPEMLALLGEEDLLLLTADHGCDPTWQGTDHTRERVPVLAYGAGL  370sp|B1KRP6|DEOB_SHEWM        K-SIVFTNFVDFDSHYGHRRDIAGYAKALEYFDSRLPEIFEILGEDDLLLLTADHGCDPSWKGTDHTRERVPVLAYGAGL  370sp|Q6LUH2|DEOB_PHOPR        N-SLVFTNFVDFDSAYGHRRNVAGYAAALEYFDKRLPEVLELLQEDDVLILTADHGCDPTWEGTDHTREHIPVIVTGPKI  372sp|B6ENG5|DEOB_ALISL        N-TIVFTNFVDFDSAYGHRRDVAGYAAALEYFDGRLPEIMEMLQEDDILILTADHGCDPTWPGTDHTREHIPVLVYGHKV  372sp|B5FAA0|DEOB_ALIFM        N-TIVFTNFVDFDSAYGHRRDVAGYAAALEYFDGRLPEIMDMLQEDDILILTADHGCDPTWPGTDHTREHIPVLVYGHKV  372sp|Q5E7J5|DEOB_ALIF1        N-TIVFTNFVDFDSAYGHRRDVAGYAAALEYFDGRLPEIMDMLQEDDILILTADHGCDPTWPGTDHTREHIPVLVYGHKV  376sp|C3LQC0|DEOB_VIBCM (2)    N-TIVFTNFVDFDSAYGHRRDVAGYAAALEYFDKRLPEVLALMQEDDILILTADHGCDPTWPGTDHTREHIPVLVYGKKV  372sp|Q9KPL9|DEOB_VIBCH        N-TIVFTNFVDFDSAYGHRRDVAGYAAALEYFDKRLPEVLELMQEDDILILTADHGCDPTWPGTDHTREHIPVLVYGKKV  372sp|A7MUW4|DEOB_VIBCB        N-TIVFTNFVDFDSAYGHRRDVAGYAAALEYFDGRINEVLEIMEEDDVLILTADHGCDPTWPGTDHTREHIPVIVYGKRV  372sp|Q7MI40|DEOB_VIBVY (2)    N-TIVFTNFVDFDSAYGHRRDVAGYAAALEYFDGRIHEVMELMQEDDILILTADHGCDPTWPGTDHTREHIPVLVYGQKV  372sp|Q87M24|DEOB_VIBPA        N-TIVFTNFVDFDSAYGHRRDVAGYAAALEYFDGRINEVLELMGEDDVLILTADHGCDPTWPGTDHTREHIPVLVYGQKV  372sp|Q2NW04|DEOB_SODGM        N-TLVFTNFVDFDSAYGHRRDVAGYAAALELFDRRLPELMSRVQGNDILILTADHGCDPTWHGTDHTREHVPVLIYGPTV  373sp|B2VH52|DEOB_ERWT9        N-SIVFTNFVDFDSAWGHRRDIPGYAAGLELFDRRLPELMALVKEGDILILTADHGCDPSWPGTEHTREHIPVLIFGPGV  373sp|A7MGA8|DEOB_CROS8        N-TIVFTNFVDFDSSWGHRRDVAGYAAGLELFDRRLPELLELVGEDDIIIFTADHGCDPTWKGTDHTREHIPVLVYGPKV  373sp|A4W6A0|DEOB_ENT38        K-TIVFTNFVDFDSSWGHRRDIAGYAAGLELFDRRLPELMELVGEDDILILTADHGCDPSWTGTDHTREHIPVLVYGPKV  373sp|Q327L3|DEOB_SHIDS        N-TIVFTNFVDFDSSWGHRRDVAGYAAGLELFDRRLPELMSLLRDDDILILTADHGCDPTWTGTDHTREHIPVLVYGPKV  373sp|Q1R260|DEOB_ECOUT (3)    N-TIVFTNFVDFDSSWGHRRDVAGYAAGLELFDRRLPELMSLLRDDDILILTADHGCDPTWTGTDHTREHIPVLVYGPKV  373sp|B7UR11|DEOB_ECO27        N-TIVFTNFVDFDSSWGHRRDVAGYAAGLELFDRRLPELMSLLRDDDILILTADHGCDPTWTGTDHTREHIPVLVYGPKV  373sp|Q3YU10|DEOB_SHISS        N-TIVFTNFVDFDSSWGHRRDVAGYAVGLELFDRRLPELMSLLRDDDILILTADHGCDPTWTGTDHTREHIPVLVYGPKV  373sp|Q0SX28|DEOB_SHIF8 (22)   N-TIVFTNFVDFDSSWGHRRDVAGYAAGLELFDRRLPELMSLLRDDDILILTADHGCDPTWTGTDHTREHIPVLVYGPKV  373sp|B5Y275|DEOB_KLEP3        E-TIVFTNFVDFDSSWGHRRDVAGYAAGLELFDRRLPELMELVGEDDILILTADHGCDPTWTGTDHTREHIPVLVYGPKV  373sp|A9MRA5|DEOB_SALAR        K-TIVFTNFVDFDSSWGHRRDIAGYASGLELFDRRLPELMALVGEDDILILTADHGCDPSWTGTDHTREHIPVLIYGPKV  373sp|B5BAJ9|DEOB_SALPK (2)    K-TIVFTNFVDFDSSWGHRRDIAGYAAGLELFDRRLPELMELVGEDDILILTADHGCDPSWTGTDHTREHIPVLIYGPKV  373sp|C0Q7M5|DEOB_SALPC (2)    K-TIVFTNFVDFDSSWGHRRDIAGYAAGLELFDRRLPELMELVGEDDILILTADHGCDPSWTGTDHTREHIPVLIYGPKV  373sp|B5R9V1|DEOB_SALG2        K-TIVFTNFVDFDSSWGHRRDIAGYAAGLELFDRRLPELMELVGEDDILILTADHGCDPSWTGTDHTREHIPVLIYGPKV  373sp|B5FTC7|DEOB_SALDC        K-TIVFTNFVDFDSSWGHRRDIAGYAAGLELFDRRLPELMELVGEDDILILTADHGCDPSWTGTDHTREHIPVLIYGPKV  373sp|P63924|DEOB_SALTI (8)    K-TIVFTNFVDFDSSWGHRRDIAGYAAGLELFDRRLPELMELVGEDDILILTADHGCDPSWTGTDHTREHIPVLIYGPKV  373sp|C5BHJ4|DEOB_EDWI9        D-TIVFTNFVDFDSSYGHRRDVAGYAAALELFDRRLPEMLALVKEDDILILTADHGCDPTWHGSDHTREHIPVLVYGPKV  373sp|Q6D990|DEOB_PECAS        N-TIVFTNFVDFDSAYGHRRDIPGYAAALELFDRRLPELMSRVTGDDILILTADHGCDPSWHGTDHTRENVPVLIYGPKV  373sp|C6DKL9|DEOB_PECCP        N-TIVFTNFVDFDSAYGHRRDIPGYAAALELFDRRLPEMLSRVKGDDILILTADHGCDPSWHGTDHTRENVPVLIYGPNV  373sp|B4EWA2|DEOB_PROMH        N-TIVFTNFVDFDSSYGHRRDVVGYGEALELFDRRLPELMELVKEDDILILTADHGCDPTWQGSDHTREHIPVLVYGPKV  374sp|Q7N931|DEOB_PHOLL        N-TIVFTNFVDFDSSYGHRRDVPGYAAALELFDRRLPEMLKRVKNDDILILTADHGCDPTWSGTDHTREHIPVLIYGPKV  373sp|A8G9H8|DEOB_SERP5        N-TIVFTNFVDFDSSYGHRRDVAGYAAALELFDRRLPELLKLVKDEDIIIFTADHGCDPTWPGTDHTREHIPVLVYGPKV  373sp|A1JJ99|DEOB_YERE8        N-TIVFTNFVDFDSSYGHRRDVAGYAAALELFDRRLPELMALVKEDDILLLTADHGCDPTWPGTDHTREHIPVLVYGPKV  373sp|A9R047|DEOB_YERPG        N-TIVFTNFVDFDSSYGHRRDVAGYAAALELFDRRLPELMALIKEDDILILTADHGCDPTWPGTDHTREHIPVLVYGPKV  373sp|A4TQJ1|DEOB_YERPP (4)    N-TIVFTNFVDFDSSYGHRRDVAGYAAALELFDRRLPELMALIKEDDILILTADHGCDPTWPGTDHTREHIPVLVYGPKV  373sp|B1JL35|DEOB_YERPY (4)    N-TIVFTNFVDFDSSYGHRRDVAGYAAALELFDRRLPELMALVKEDDILILTADHGCDPTWPGTDHTREHIPVLVYGPKV  373Consensus                   KPG--SLGRRET----FADIGQTIASYFGL-SPMDYGKSFLNFKGQPXSSHASTTGD                         423deoB_Bcereus_WP_098782964.1 KEGGQELPLRQT----FADIGATVAENFGV-KMPEYGKSFLNELKK-----------                         394deoB_Rel606                 KPG--SLGHRET----FADIGQTLAKYFGT-SDMEYGKAMF----------------                         407sp|Q89A57|DEOB_BUCBP        ESK--NFGYRET----FSDIGQTLAKYFNL-STMSYGTSIF----------------                         408sp|Q8K936|DEOB_BUCAP        EIK--FLGHRDT----FSDIGQTIAKYFSL-SDMKYGKSMF----------------                         409sp|B8D866|DEOB_BUCAT        KKN--FLGHRKT----FADIGQTIAKYFLL-TDMSYGQNML----------------                         407sp|P57607|DEOB_BUCAI        KKN--FLGHRKT----FADIGQTIAKYFLL-TDMSYGQNML----------------                         407sp|B8D9W4|DEOB_BUCA5        KKN--FLGHRKT----FADIGQTIAKYFLL-TDMSYGQNML----------------                         407sp|Q483R0|DEOB_COLP3        DSV--NLGNRKT----FADLGQSVAELFNV-EAMDYGTSFLSEIYSK----------                         404sp|Q3ICU9|DEOB_PSET1        TDI--PLGERNS----FADIGQTLAQWFNL-PALEYGDGFIDKLTTSK---------                         405sp|Q5QXT9|DEOB_IDILO        QAG--NRGKRHS----FADLGQTLCRLFDL-PAMEEGKAIKLS--------------                         399sp|Q17YP1|DEOB_HELAH        QPT--FLGRSET----FADIGQSIAHFLGL-SPLDYGKNLLNFKGQP----------                         413sp|Q9ZK37|DEOB_HELPJ        QPA--FLGKSET----FADIGQSIAYFLGL-SPLDYGKNLLNFKGQP----------                         413sp|B6JN18|DEOB_HELP2        QPA--FLGKSDS----FADIGQSIAYFLGL-SPLDYGKNLLNFKGQP----------                         413sp|P56195|DEOB_HELPY        QPA--FLGKSES----FADIGQSIAHFLGL-SPLDYGKNLLNFKGQP----------                         413sp|B5Z8H3|DEOB_HELPG        QPA--FLGKSES----FADIGQSIAYFLGL-SPLDYGKNLLKFKGQP----------                         413sp|B2UUU2|DEOB_HELPS        QPA--FLGKSES----FADIGQSIAYFLGL-SPLDYGKNLLNFKGQS----------                         413sp|Q1CS87|DEOB_HELPH        QPA--FLGKSES----FADIGQSIAYFLGL-SPLDYGKNLLNFKGQP----------                         413sp|A4YPV1|DEOB_BRASO        SAG--SIGARTS----FADTGATIAQHLQL-PPTRHGNSFWPNGA------------                         407sp|B8IJW7|DEOB_METNO        RPG--PLGRRDT----FADIGAALAAHLGL-PP-SCGRSWL----------------                         407sp|B0UPC1|DEOB_METS4        RPG--PIGRREG----LADIGATVAAHLGL--ALPAGRSWL----------------                         405sp|B1LYJ9|DEOB_METRJ        AAR--PIGRRET----FADIGATVAAHLGL-AWDGAGTPFL----------------                         406sp|B1ZIG8|DEOB_METPB        ATG--AIGRRET----FADIGASVAAHLGL-PPLGAGRAWW----------------                         406sp|A9W427|DEOB_METEP        APR--ALGRRES----FADMGASVAAHLGL-PPLGAGQAWW----------------                         406sp|B7KXI3|DEOB_METC4        TPR--ALGRRES----FADMGASVAAHLGL-PPLGAGQAW-----------------                         405sp|C3MBH5|DEOB_SINFN        RSR--SFGIANS----FAHIGETAARHLGI-APGPHGRSLL----------------                         406sp|Q92T47|DEOB_RHIME        RSR--SFGIADS----FAHIGETVARHLGI-GVGPHGRSLI----------------                         406sp|A6UET6|DEOB_SINMW        RSR--SVGIVGS----FAHIGETVASHLGI-DPGPHGRSLI----------------                         406sp|B9JYP7|DEOB_AGRVS        RAR--SIGIRDT----YADIGETIAAHLGI-APGRHGMSFL----------------                         406sp|Q8UJ04|DEOB_AGRFC        RSR--DVGIRSS----YADIGESIAHHLGI-EAGSHGRSFI----------------                         406sp|B9J6V9|DEOB_AGRRK        RSR--PIGIRQT----YADIGETVAHHLGI-AAGPHGRSFL----------------                         406sp|Q2KDR4|DEOB_RHIEC        RSR--SIGVRRT----YADIGESIARHLGI-PAGPHGRSFL----------------                         406sp|B5ZXI4|DEOB_RHILW        RSR--SIGVRRS----YADIGESIARHLGI-PAGPHGRSFL----------------                         406sp|Q1MMV6|DEOB_RHIL3        RSR--SIGVRRG----YADIGESIARHLGI-PAGPHGRSFL----------------                         406sp|Q98BG5|DEOB_RHILO        AGG--DIGLRTT----FADIGETVAEHLGL-APGRHGTSFHAMIGGHA---------                         413sp|Q11AV9|DEOB_CHESB        AKG--SIGRRRS----YADIGETIASHLAL-PAGRHGVSFLNALQHA----------                         409sp|A5IGR8|DEOB_LEGPC        NSK--FIGRRDC----FADIGQSIAEHLQLSSPLAHGVSFL----------------                         407sp|Q5X7B2|DEOB_LEGPA        NSK--FIGRRDC----FADIGQSIAEHLQLSSPLTHGVSFL----------------                         407sp|Q5WYR0|DEOB_LEGPL        NSK--FIGRRDC----FADIGQSIAEHLQLSSPLTHGVSFL----------------                         407sp|Q5ZXU2|DEOB_LEGPH        NSK--FIGRRDC----FADIGQSIAEHLQLSSPLAHGVSFL----------------                         407sp|B2KBT7|DEOB_ELUMP        KNG--FIGGRET----YSDLGQTVAEYLGI-TKLNNGTSFL----------------                         397sp|Q2SHN3|DEOB_HAHCH        PHG--FIGARKT----FADIGQTLAEYFQL-DRLDFGESFLVADK------------                         414sp|Q1QZC2|DEOB_CHRSD        APR--PLGHDDGHLVTFADIGQSLATHFGL-SPMAHGSDFLDVAPTGPSSHASTTGD                         418sp|A1SYK3|DEOB_PSYIN        KPG--PIGLRET----FADIGQSIADFHKL-PALAYGKSIFS---------------                         415sp|A6VM02|DEOB_ACTSZ        PAR--FLGARET----FADIGQTVAKYLGV-SPMEYGTAII----------------                         395sp|C4LAY9|DEOB_TOLAT        QPQ--DLGMRDT----FADIGQTIAAYHGL-PVLDYGSNCLPEHH------------                         408sp|A4SRU2|DEOB_AERS4        KAG--SVGRRET----FADIGQSIAAYHGL-PKLEYGTSFL----------------                         402sp|A0KPE2|DEOB_AERHH        KAG--SVGRRET----FADIGQSIAAYHGL-PKLAYGTSFL----------------                         402sp|Q7NRT1|DEOB_CHRVO        KAG--PIGERAT----FADIGQSVAAHLGL-PRMDYGTSFLD---------------                         405sp|Q086F8|DEOB_SHEFN        SAG--SLGRRKS----FADIGQSIASYFKL-EPMNYGESFIN---------------                         405sp|Q12QG1|DEOB_SHEDO        AAG--SLGLRNS----FADMGQSIASYFEL-EPMEYGESFIQ---------------                         405sp|A1S476|DEOB_SHEAM        EAG--SLGKRGS----FADIGQSIASYFGL-AAMEYGESFMPAR-------------                         407sp|Q8EHK2|DEOB_SHEON        KAG--SLGRRNS----FADIGQSIASYFKL-EPMEYGESFI----------------                         404sp|A0KU09|DEOB_SHESA        KAG--SLGRRNS----FADIGQSIASYFKL-EPMEYGESFI----------------                         404sp|Q0HLE8|DEOB_SHESM        KAG--SLGRRNS----FADIGQSIASYFKL-EPMEYGESFI----------------                         404sp|Q0HXQ2|DEOB_SHESR        KAG--SLGRRNS----FADIGQSIASYFKL-EPMEYGESFI----------------                         404sp|A1RH89|DEOB_SHESW        KAG--SLGRRNS----FADIGQSIASHFKL-EPMAYGESFI----------------                         404sp|A4Y9A6|DEOB_SHEPC        KAG--SLGRRNS----FADIGQSIASHFKL-EPMAYGESFI----------------                         404sp|A6WRB6|DEOB_SHEB8        KAG--SLGRRKS----FADIGQSIASHFKL-EPMAYGESFL----------------                         404sp|A9KZ78|DEOB_SHEB9 (2)    KAG--SLGRRKS----FADIGQSIASHFKL-EPMAYGESFL----------------                         404sp|B8E6P6|DEOB_SHEB2        KAG--SLGRRKS----FADIGQSIASHFKL-EPMAYGESFL----------------                         404sp|B0TQ89|DEOB_SHEHH        EPG--SLGLRNS----FADMGQSIASYHKL-EPMEYGESFVRS--------------                         406sp|A8H726|DEOB_SHEPA        EAG--SLGLRSS----FADMGQSIASYFKL-EPMEYGESFIR---------------                         405sp|A8FYQ7|DEOB_SHESH        EAG--SLGRRKS----FADMGQSIASYFKL-EPMEYGESFIK---------------                         405sp|A3QGT1|DEOB_SHELP        APG--SLGRRNS----FADIGQSIASYFGL-EPMEYGESFVA---------------                         405sp|B1KRP6|DEOB_SHEWM        KAG--SLGRRNS----FADIGQSIASYFKL-EPMEYGESFIK---------------                         405sp|Q6LUH2|DEOB_PHOPR        PAG--SLGRRET----FADIGQSLAEYYGT-SDMEYGKSFL----------------                         406sp|B6ENG5|DEOB_ALISL        PAG--SLGLRDT----FADIGQTLAEYFEI-SDMEYGKSFL----------------                         406sp|B5FAA0|DEOB_ALIFM        PAG--SLGRRDT----FADIGQTLAEYFET-SDMEYGKSFL----------------                         406sp|Q5E7J5|DEOB_ALIF1        PAG--SLGRRDT----FADIGQTLAEYFET-SDMEYGKSFL----------------                         410sp|C3LQC0|DEOB_VIBCM (2)    APG--SLGRRDT----FADIGQTLASYFGT-SPMDYGKNFL----------------                         406sp|Q9KPL9|DEOB_VIBCH        APG--SLGRRDT----FADIGQTLASYFGT-SPMDYGKNFL----------------                         406sp|A7MUW4|DEOB_VIBCB        PAG--SLGRRDS----FADIGQTLATYFGT-SPMDYGKNFL----------------                         406sp|Q7MI40|DEOB_VIBVY (2)    PAG--SLGRRET----FADIGQTLASYFGT-SPMDYGKNFL----------------                         406sp|Q87M24|DEOB_VIBPA        PAG--SLGRRET----FADIGQTLASYFGT-SPMDYGKNFL----------------                         406sp|Q2NW04|DEOB_SODGM        NPG--FYGHRTT----FADIGQTVARYFGL-SPMDYGKAIM----------------                         407sp|B2VH52|DEOB_ERWT9        RPG--DYGYRDT----FADIGQTLAHYFGL-SPMAYGKPFF----------------                         407sp|A7MGA8|DEOB_CROS8        KPG--SLGHRDT----FADIGQTVAKYFGL-SDMEYGKALF----------------                         407sp|A4W6A0|DEOB_ENT38        KPG--SLGHRET----FADIGQTIASYFGT-SPMDYGKNML----------------                         407sp|Q327L3|DEOB_SHIDS        KPG--SLGHRET----FADIGQTLAKYFGT-SDMEYGKAMF----------------                         407sp|Q1R260|DEOB_ECOUT (3)    KPG--SLGHRET----FADIGQTLAKYFGT-SDMEYGKAMF----------------                         407sp|B7UR11|DEOB_ECO27        KPG--SLGHRET----FADIGQTLAKYFGT-SDMEYGKAMF----------------                         407sp|Q3YU10|DEOB_SHISS        KPG--SLGHRET----FADIGQTLAKYFGT-SDMEYGKAMF----------------                         407sp|Q0SX28|DEOB_SHIF8 (22)   KPG--SLGHRET----FADIGQTLAKYFGT-SDMEYGKAMF----------------                         407sp|B5Y275|DEOB_KLEP3        KPG--SLGHRET----FADIGQTIAKYFGT-SDMEYGKAMF----------------                         407sp|A9MRA5|DEOB_SALAR        KPG--SLGHRET----FADIGQTLASYFGT-SPMDYGKNML----------------                         407sp|B5BAJ9|DEOB_SALPK (2)    KPG--SLGHRET----FADIGQTLATYFGT-SPMDYGKNML----------------                         407sp|C0Q7M5|DEOB_SALPC (2)    KPG--SLGHRET----FADIGQTLATYFGT-SPMDYGKNML----------------                         407sp|B5R9V1|DEOB_SALG2        KPG--SLGHRET----FADIGQTLATYFGT-SPMDYGKNML----------------                         407sp|B5FTC7|DEOB_SALDC        KPG--SLGHRET----FADIGQTLATYFGT-SPMDYGKNML----------------                         407sp|P63924|DEOB_SALTI (8)    KPG--SLGHRET----FADIGQTLATYFGT-SPMDYGKNML----------------                         407sp|C5BHJ4|DEOB_EDWI9        KPG--SLGERDT----FADIGQTVARYFGL-SPMAYGKPMF----------------                         407sp|Q6D990|DEOB_PECAS        KPG--SYGHRET----FADIGQTVAAYFGL-SPMDYGKSIL----------------                         407sp|C6DKL9|DEOB_PECCP        KPG--SYGHRET----FADIGQTVAAYFGL-SPMDYGKSIL----------------                         407sp|B4EWA2|DEOB_PROMH        KPG--SLGHRET----FADIGQTVVKYFGL-SPVEYGKAMF----------------                         408sp|Q7N931|DEOB_PHOLL        QPG--SLGHRET----FADIGQTVAKYFEL-SPMEYGKSML----------------                         407sp|A8G9H8|DEOB_SERP5        KPG--SLGHRET----FADIGQTVASYFGV-SPMDYGKSMF----------------                         407sp|A1JJ99|DEOB_YERE8        KPG--SLGHRET----FADIGQTVAKYFDL-SPMDYGKNML----------------                         407sp|A9R047|DEOB_YERPG        KPG--SLGHRET----FADIGQTVAAYFGL-SPMDYGKNML----------------                         407sp|A4TQJ1|DEOB_YERPP (4)    KPG--SLGHRET----FADIGQTVAAYFGL-SPMDYGKNML----------------                         407sp|B1JL35|DEOB_YERPY (4)    KPG--SLGHRET----FADIGQTVAAYFGL-SPMDYGKNML----------------                         407
